# SIRT6 Lysine‐Demyristoylates ATF2 to Ameliorate Vascular Injury via PRKCD/VE‐Cadherin Pathway Regulating Vascular Endothelial Barrier

**DOI:** 10.1002/advs.202504948

**Published:** 2025-08-14

**Authors:** Runyang Feng, Zheyan Fang, Shuang Zhao, You‐en Zhang, Na Wu, Zhenyang Guo, Xin Zhong, Bixuan Jiang, Hongfei Xu, Hangnan Hong, Zhentao Zhang, Mukaddas Abdurahman, Xueting Yu, Jilong Geng, Xiansu Nie, Supuya Abuduwahapi, Dong Huang, Gang Zhao, Wenbin Zhang, Hao Lu, Li Shen, Xin Zhao, Zhaoyang Chen, Junjie Guo, Hongchao Zheng, Yue He, Sanwu Wu, Jiayin Peng, Jiawen Song, Xiao Wang, Haomin Li, Yuli Huang, Shengying Qin, Haojie Lu, Xiangdong Wang, Jianguo Zhao, Lei Huang, Yun Zhao, Peng Li, Junbo Ge, Hua Li

**Affiliations:** ^1^ Department of Cardiology Zhongshan Hospital Shanghai Institute of Cardiovascular Diseases Fudan University Shanghai 200032 China; ^2^ Department of Medical Examination Shanghai Xuhui District Central Hospital Shanghai 200031 China; ^3^ Department of Cardiology Arteriosclerosis Cardiovascular Disease Clinical Medical Research Center of Hubei Province Renmin Hospital Hubei University of Medicine Shiyan Hubei 442000 China; ^4^ Department of Cardiology Shanghai Xuhui District Central Hospital Shanghai 200031 China; ^5^ Bio‐X Institutes Key Laboratory for the Genetics of Developmental and Neuropsychiatric Disorders (Ministry of Education) Shanghai Jiao Tong University Shanghai 200030 China; ^6^ School of Life Sciences and Biotechnology Shanghai Jiao Tong University Shanghai 200240 China; ^7^ Department of Forensic Medicine Soochow University Suzhou 215000 China; ^8^ Department of Cardiology Sir Run Run Shaw Hospital affiliated with Zhejiang University School of Medicine Hangzhou 310020 China; ^9^ Department of Cardiology Heart Center of Fujian Province Fujian Medical University Union Hospital Fuzhou 350001 China; ^10^ Department of Cardiology The Affiliated Hospital of Qingdao University Qingdao 266003 China; ^11^ Department of Cardiology Shanghai Eighth People's Hospital Shanghai 200235 China; ^12^ State Key Laboratory of Cell Biology Shanghai Institute of Biochemistry and Cell Biology Center for Excellence in Molecular Cell Science Chinese Academy of Sciences University of Chinese Academy of Sciences Shanghai 200031 China; ^13^ State Key Laboratory of Animal Nutrition Institute of Animal Science Chinese Academy of Agricultural Sciences Beijing 100193 China; ^14^ State Key Laboratory of Stem Cell and Reproductive Biology Institute of Zoology Chinese Academy of Sciences Beijing 100101 China; ^15^ Clinical Data Center the Children's Hospital, Zhejiang University School of Medicine National Clinical Research Center for Child Health Hangzhou Zhejiang 310052 China; ^16^ Department of Cardiology Shunde Hospital Southern Medical University Guangdong 528399 China; ^17^ Department of Chemistry and Institutes of Biomedical Sciences Fudan University Shanghai 200032 China; ^18^ Department of Pulmonary and Critical Care Medicine Zhongshan Hospital Shanghai Medical College Fudan University Shanghai 200032 China; ^19^ Savaid Medical School University of Chinese Academy of Sciences Beijing 100049 China; ^20^ Beijing Institute for Stem Cell and Regenerative Medicine Beijing 100101 China; ^21^ Department of Molecular Cell and Cancer Biology University of Massachusetts Chan Medical School Worcester MA 01605 USA; ^22^ School of Life Science and Technology ShanghaiTech University 100 Haike Road Shanghai 201210 China; ^23^ Key Laboratory of Systems Health Science of Zhejiang Province School of Life Science Hangzhou Institute for Advanced Study University of Chinese Academy of Sciences Hangzhou 310024 China; ^24^ National Basic Science Center for Complex System of Panvascular Intervention Shanghai 200032 China; ^25^ National Clinical Research Center for Interventional Medicine Shanghai 200032 China; ^26^ Institutes of Biomedical Sciences Fudan University Shanghai 200032 China; ^27^ Shanghai Clinical Research Center for Interventional Medicine Shanghai 200032 China; ^28^ Key Laboratory of Viral Heart Diseases National Health Commission Shanghai 200032 China; ^29^ Key Laboratory of Viral Heart Diseases Chinese Academy of Medical Sciences Shanghai 200032 China

**Keywords:** ATF2, endothelial barrier function, epigenetic regulation, myristoylation, nucleoplasmic translocation, SIRT6

## Abstract

Cardiovascular diseases (CVDs) progression is significantly modulated by epigenetic mechanisms, particularly through Sirtuin 6 (SIRT6), a key NAD⁺‐dependent deacetylase in the sirtuin family. Though essential for cardiovascular homeostasis, the effects of SIRT6‐mediated lysine myristoylation on CVDs progression remain largely unexplored due to detection limitations. This study developes an innovative lysine‐myristoylated peptide enrichment technique, identifying mutant SIRT6 (H133Y) with high myristoyl affinity but deficient demyristoylase activity. This advancement enables identification of 15 previously unrecognized human lysine‐myristoylated proteins. Further study demonstrates that SIRT6 demyristoylates activating transcription factor 2 (ATF2) at K296 and regulates its nucleoplasmic translocation. Through 4D label‐free mass spectrometry and molecular approaches, it is revealed that decreased nuclear localization of ATF2 results in reduced Protein kinase C delta type (PRKCD) expression, establishing a SIRT6/Myr‐ATF2/PRKCD/VE‐Cadherin pathway that enhances endothelial barrier integrity under high myristate conditions. These findings are validated in vitro (gene overexpression/knockdown cells) and in vivo (SIRT6 knockout/double‐transgenic mice). The study provides both a novel method for identifying lysine‐myristoylated proteins and critical insights into SIRT6 demyristoylation biology. Modulating the SIRT6 pathway might yield therapies to strengthen endothelial integrity and mitigate vascular dysfunction in CVDs, offering promising clinical translation avenues.

## Graphical abstract

A novel modified SIRT6 variant (dSIRT6) H133Y‐mediated enrichment technique unmasks 15 new human lysine‐myristoylated proteins. Notably, Sirtuin 6 (SIRT6) demyristoylation of activating transcription factor 2 (ATF2) at K296 orchestrates its nucleoplasmic translocation. Moreover, the SIRT6/Myr‐ATF2/ Protein kinase C delta type (PRKCD)/VE‐Cadherin axis uncovers an innovative epigenetic mechanism governing endothelial barrier function. Applicationally, this work reveals that SIRT6‐mediated demyristoylation of ATF2 strengthening endothelial barrier integrity provides promising clinical targets for improving cardiovascular health.

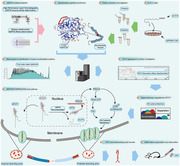

## 1 Introduction

In the intricate realm of human health, vascular system homeostasis is paramount. Vascular permeability constitutes a critical regulatory mechanism governing the exchange of fluids, nutrients, and signaling molecules between circulation and interstitial. Dysregulation of this process is implicated in diverse pathologies including stroke, sepsis, diabetic vascular complications, acute lung injury, and malignancies tissues.^[^ ^1^, ^2^, ^3^, ^4^ ^]^ Endothelial cells maintain this semipermeable barrier between vascular lumen and surrounding tissues, while endothelial dysfunction manifests as impaired barrier integrity and increased permeability, contributing to vascular homeostasis disruption.^[^ ^5^ ^]^ Chronic inflammation, oxidative stress, and impaired signaling pathways drive this dysfunction, accelerating Cardiovascular diseases (CVDs) progression.^[^ ^6^, ^7^ ^]^

Emerging evidence positions epigenetic modifications, particularly those mediated by chromatin‐modifying enzymes, as central regulators of cardiovascular biology. Histone methyltransferases (HMTs), deacetylases (HDACs), and acetyltransferases (HATs) modulate key processes in hypertension, heart failure, and cardiac hypertrophy pathogenesis.^[^ ^8^ ^]^ Among sirtuins (SIRTs), the NAD⁺‐dependent deacetylase Sirtuin 6 (SIRT6) has garnered significant attention for its roles in cardiovascular physiology and pathology.^[^ ^9^, ^10^ ^]^ SIRT6 promotes DNA repair, genome stabilization, and metabolic homeostasis,^[^ ^11^ ^]^ with demonstrated protective effects against inflammation and oxidative stress in ischemic stroke and myocardial infarction, both of which are critical factors in vascular dysfunction. Nevertheless, despite the growing volume of research, the involvement of SIRT6 in endothelial barrier function has largely remained unexamined. Specifically, its role in defatty‐acylation, and their impact on vascular permeability have yet to be fully elucidated. Protein lysine fatty acylation (KFA) represents a post‐translational modification with significant relevance to multiple biological processes.^[^ ^12^, ^13^, ^14^ ^]^ This modification involves the attachment of long‐chain fatty acyl groups to lysine residues.^[^ ^15^ ^]^ Notably, KFA is reversible, with specific acylhydrolases—including SIRT and HDAC family members,^[^ ^16^, ^17^, ^18^, ^19^, ^20^ ^]^ mediating fatty acyl group removal. These modifications critically regulate protein‐protein interactions, membrane localization, and protein secretion.^[^ ^18^, ^21^, ^22^, ^23^ ^]^ However the regulatory mechanisms governing lysine fatty acylation in endothelial contexts remain poorly understood.

Among endothelial transcription factors, activating transcription factor 2 (ATF2) plays a prominent role in regulating apoptosis, inflammation, angiogenesis, and atherosclerosis.^[^ ^24^, ^25^, ^26^, ^27^, ^28^ ^]^ Although ATF2 exhibits strict transcriptional regulation of endothelial genes, myristoylation's impact on its barrier integrity function remains unexplored. Technical limitations primarily hinder investigation of lysine myristoylation in endothelial biology.^[^ ^29^ ^]^ Traditional detection methods relying on peptide enrichment with mass spectrometry (MS),^[^ ^30^, ^31^ ^]^ which face significant constraints, including: absence of myristoyl‐lysine specific antibodies, and potential false positives from glycosylphosphatidylinositol (GPI)‐anchored proteins in click chemistry approaches. These challenges have restricted comprehensive understanding of lysine myristoylation's physiological scope.

To overcome these limitations, we developed a novel enrichment methodology centered on a modified SIRT6 variant (dSIRT6). This approach enabled the identification of 15 previously unrecognized lysine‐myristoylated proteins. We subsequently characterized the functional consequences of SIRT6‐mediated demyristoylation through comprehensive in vitro and in vivo investigations, elucidating their contributions to endothelial function and vascular permeability. This work aims to: delineate molecular mechanisms underlying SIRT6‐mediated endothelial barrier regulation; establish novel insights into post‐translational modifications governing vascular homeostasis; define SIRT6's role in barrier stabilization via demyristoylation; determine myristoylation's impact on ATF2 functionality, and; resolve technical barriers in lysine myristoylation research to advance CVDs understanding.

## 2 Results

### 2.1 Identification of H133Y as dSIRT6 for Enhanced Myristoylated Peptide Enrichment

**Figure 1:**
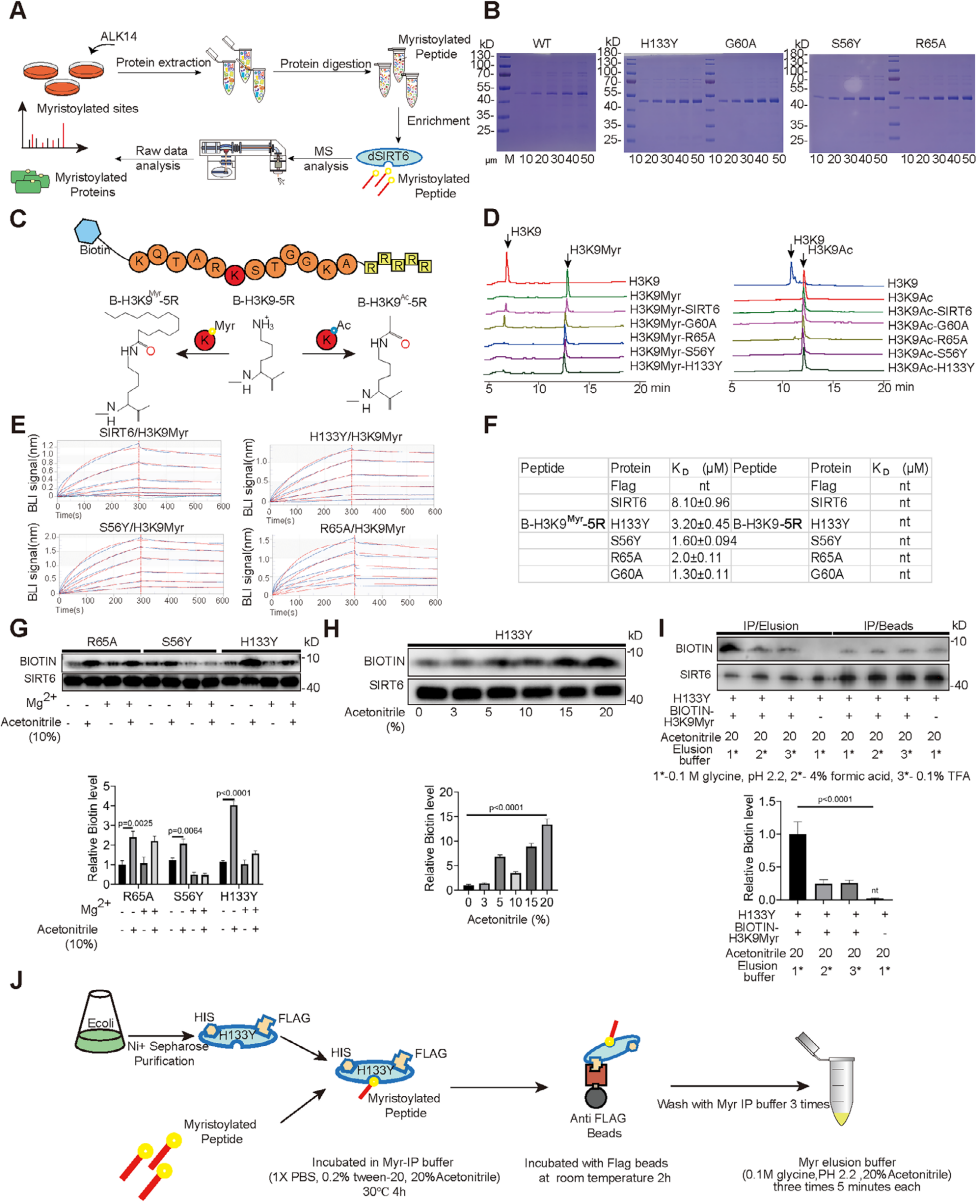
Identification and characterization of dSIRT6 mutants for myristoylated peptide enrichment. A) The procedure for enriching myristoylated peptides using dSIRT6 for mass spectrometry (MS) analysis, highlighting the experimental setup. B) Analysis of purified SIRT6 and its mutants (H133Y, G60A, S56Y, R65A) across a concentration gradient, using SDS‐PAGE and Coomassie Blue Staining to evaluate their purity and concentration. C) Design of biotinylated and arginine‐rich peptides (B‐H3K9‐5R, B‐H3K9^Myr^‐5R, B‐H3K9Ac‐5R) for the study of SIRT6 binding and activity. D) Utilizes High‐Performance Liquid Chromatography (HPLC) to assess the deacetylase and demyristoylase activities of SIRT6 mutants, providing insight into their enzymatic functions. E) Employs Biolayer Interferometry (BLI) to quantify the binding affinity of SIRT6 and its mutants to myristoylated peptides, demonstrating the specificity and strength of interactions. F) Compiles affinity data for the interaction of SIRT6 and its mutants with myristoylated and non‐myristoylated peptides, summarizing the specificity of binding. G,H) Illustrates the use of Co‐Immunoprecipitation (Co‐IP) for optimizing the enrichment of myristoylated peptides by dSIRT6, including system selection and reaction conditions. I) The optimization of elution conditions for myristoylated peptide enrichment, using acetonitrile to enhance recovery. J) Schematic overview of the H133Y‐based enrichment method for myristoylated peptides, detailing the procedure and its applications.

Previous studies on SIRT6 mutants (G60A, H133Y, S56Y, and R65A) have confirmed their pivotal roles in deacetylase, ribosyltransferase, and demyristoylase enzymatic activities.^[^ ^21^ ^]^ In this study, these mutants were purified to >90% purity, with each tagged with a His‐tag at the N‐terminus and a Flag‐tag at the C‐terminus, enabling their use as potential dSIRT6 candidates for myristoylated peptide enrichment (**Figure** **1**A,B). Additionally, H3K9 peptides modified at the C‐terminus with arginine residues and at the N‐terminus with biotin were synthesized in both acetylated and myristoylated forms, designated as B‐H3K9‐5R, B‐H3K9^Ac^‐5R, and B‐H3K9^Myr^‐5R (Figure 1C). Subsequent High‐Performance Liquid Chromatography (HPLC) analysis revealed distinct enzymatic activities among the mutants. Wild‐type SIRT6 (SIRT6 WT) exhibited significant demyristoylase activity but weak deacetylase activity. The G60A mutant showed strong demyristoylase activity without detectable deacetylase activity, whereas R65A lacked demyristoylase activity but displayed weak deacetylase activity. H133Y and S56Y mutants lacked both enzymatic activities (Figure 1D). Based on these profiles, H133Y, S56Y, and R65A were selected for myristoylated peptide enrichment. Biolayer interferometry (BLI) assays quantified binding affinities of dSIRT6 variants and SIRT6 for the myristoylated peptide B‐H3K9^Myr^‐5R. SIRT6 demonstrated high affinity (KD = 8.10 ± 0.96 µm), while dSIRT6 variants exhibited moderately higher affinities (KD range: 1.19–3.65 µM), confirming specific binding to myristoylated peptides (Figure 1E,F; Figure S1, Supporting Information).

Further optimization of reaction conditions through BLI assays with various cofactors (Mg^2+^, Zn^2+^, ATP, ADP, cAMP, AMP, NAD^+^) demonstrated that Mg^2+^, ATP, or ADP significantly enhanced SIRT6 affinity for B‐H3K9^Myr^‐5R, with Mg^2+^ alone yielding the highest affinity (Figures S2,S3A,B, Supporting Information). This pattern was consistent for dSIRT6 variants, particularly H133Y, which exhibited the highest affinity in the presence of Mg^2+^. Co‐immunoprecipitation (Co‐IP) assays identified H133Y as the optimal dSIRT6 variant for enrichment, showing the strongest interaction with B‐H3K9^Myr^‐5R in Mg^2^⁺‐free systems containing 10% acetonitrile (Figure 1G). The Myr‐IP buffer (1× PBS with 0.2% Tween‐20) was optimized to 20% acetonitrile concentration to maximize H133Y–B‐H3K9^Myr^‐5R binding (Figure 1H). Meanwhile, the negative control further confirmed the selectivity of H133Y for myristoylated peptide (Figure S3C, Supporting Information). Elution efficiency testing revealed that 0.1 m glycine (pH 2.2) with 20% acetonitrile provided optimal myristoylated peptide recovery (Figure 1I). Collectively, this study established a novel myristoylated peptide enrichment method through identification of the critical H133Y dSIRT6 variant and optimization of reaction/elution buffers (Figure 1J)

### 2.2 Identification of Lysine Myristoylated Proteins in 293T Cells Using the H133Y‐Based Peptide Enrichment Method

**Figure 2:**
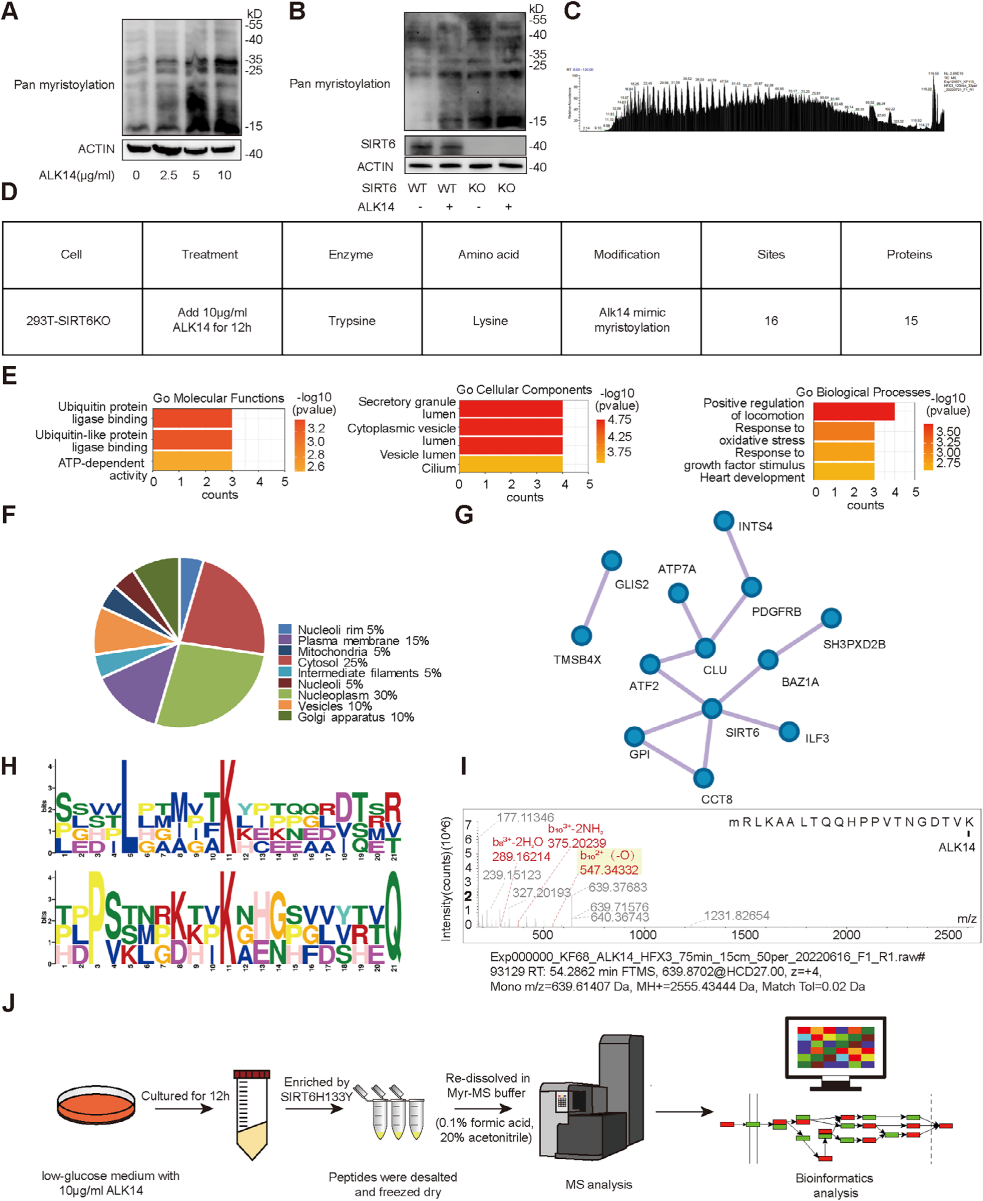
Exploration of lysine myristoylation in SIRT6 KO Cells. A,B) Investigate global myristoylation levels in SIRT6 knockout (KO) 293T cells, using western blot analysis to determine the effects of ALK14 treatment across a concentration gradient. C) A mass spectrum of peptides enriched by the H133Y mutant, highlighting the precision of the enrichment method. D) The conditions and results from MS analyses, providing data on the identification process. E) Features Gene Ontology (GO) enrichment analysis of identified myristoylated proteins, indicating their biological significance and functional categories. F) The subcellular localization of identified myristoylated proteins, offering insights into their roles within the cell. G) Depicts a protein interaction network centered on SIRT6, illustrating the connections and potential regulatory mechanisms involving myristoylated proteins. H) Analyzes identified myristoylation motifs using bioinformatics tools, contributing to the understanding of myristoylation patterns and preferences. I) A mass spectrum of ATF2 highlighting a specific myristoylation site, exemplifying the method's ability to pinpoint post‐translational modifications. J) Outlines the comprehensive approach of combining H133Y‐based immunoprecipitation with mass spectrometry (IP‐MS) for the identification of myristoylated proteins in cells treated with ALK14, elucidating the methodological framework and its application to studying protein myristoylation.

Next, we employed the H133Y‐based peptide enrichment method to identify lysine‐myristoylated proteins in 293T cells. ALK14^[^ ^32^ ^]^ (an alkyne‐modified palmitic acid analog) was introduced into the culture medium to mimic myristic acid. Western blot analysis demonstrated dose‐dependent increases in global myristoylation levels across ALK14 concentrations (0–10 µg mL^−1^) (**Figures** **2**A,S4A, Supporting Information). Comparative analysis of SIRT6 WT versus SIRT6 knockout (KO) cells, with or without ALK14 treatment, revealed significantly elevated global myristoylation levels in both SIRT6 KO cells and ALK14‐treated groups, confirming SIRT6's demyristoylase activity under these conditions (Figure 2B; Figure S4B, Supporting Information).

Subsequent experiments utilized SIRT6 KO 293T cells cultured for 12 h in low‐glucose medium supplemented with 10 µg/ml ALK14. Following collection, cells were trypsin‐digested into peptides, desalted, and lyophilized. Peptides were reconstituted in Myr‐MS buffer (0.1% formic acid, 20% acetonitrile), incubated with dSIRT6 (H133Y) for FLAG‐bead CO‐IP, and eluted for MS analysis. This identified 15 lysine‐ALK14‐labeled proteins (myristoylation mimics) were exhibited with characteristic mass spectra (Figure 2C,D and Table S1, Supporting Information). Gene Ontology (GO), UniProt, and protein interaction network analyses revealed that these proteins are primarily involved in cellular response pathways, localized to secretory granules/vesicles, and exhibit ubiquitin protein ligase binding activity (Figure 2E). UniProt annotations confirmed enrichment in protein secretion components (Golgi, vesicles, plasma membrane) (Figure 2F). Network analysis highlighted 6 lysine‐myristoylated proteins with strong SIRT6 interactions (Figure 2G), suggesting a potential direct targeting. The sequence motif analysis using the Motif‐X program identified two conserved motifs around the K^Myr^ sites, specifically L#####K^Myr^ and P#######K^Myr^#########Q (Figure 2H). To select myristoylated proteins directly targeted by SIRT6, we selected CLU, GPI, CCT8, IF3, BAZ1A, and ATF2 based on PPI results. Considering that SIRT6 is mainly located in the nucleus, suggesting the nuclear localization of its substrates, we further screened BAZ1A and ATF2, which have nuclear localization. Finally, ATF2, rather than BAZ1A, located in a common motif (P#######K^Myr^#########Q) identified by IP‐MS (Figure 2I), making it a primary candidate for further validation of the H133Y enrichment method and exploration of its biological processes of post‐myristoylation. Collectively, the H133Y‐based method identified 15 lysine‐myristoylated proteins in cultured cells, with ATF2 emerging for further investigation, demonstrating the approach's utility in profiling lysine myristoylation (Figure 2J).

### 2.3 Biological Function of Myristoylation at K296 Site of ATF2

**Figure 3:**
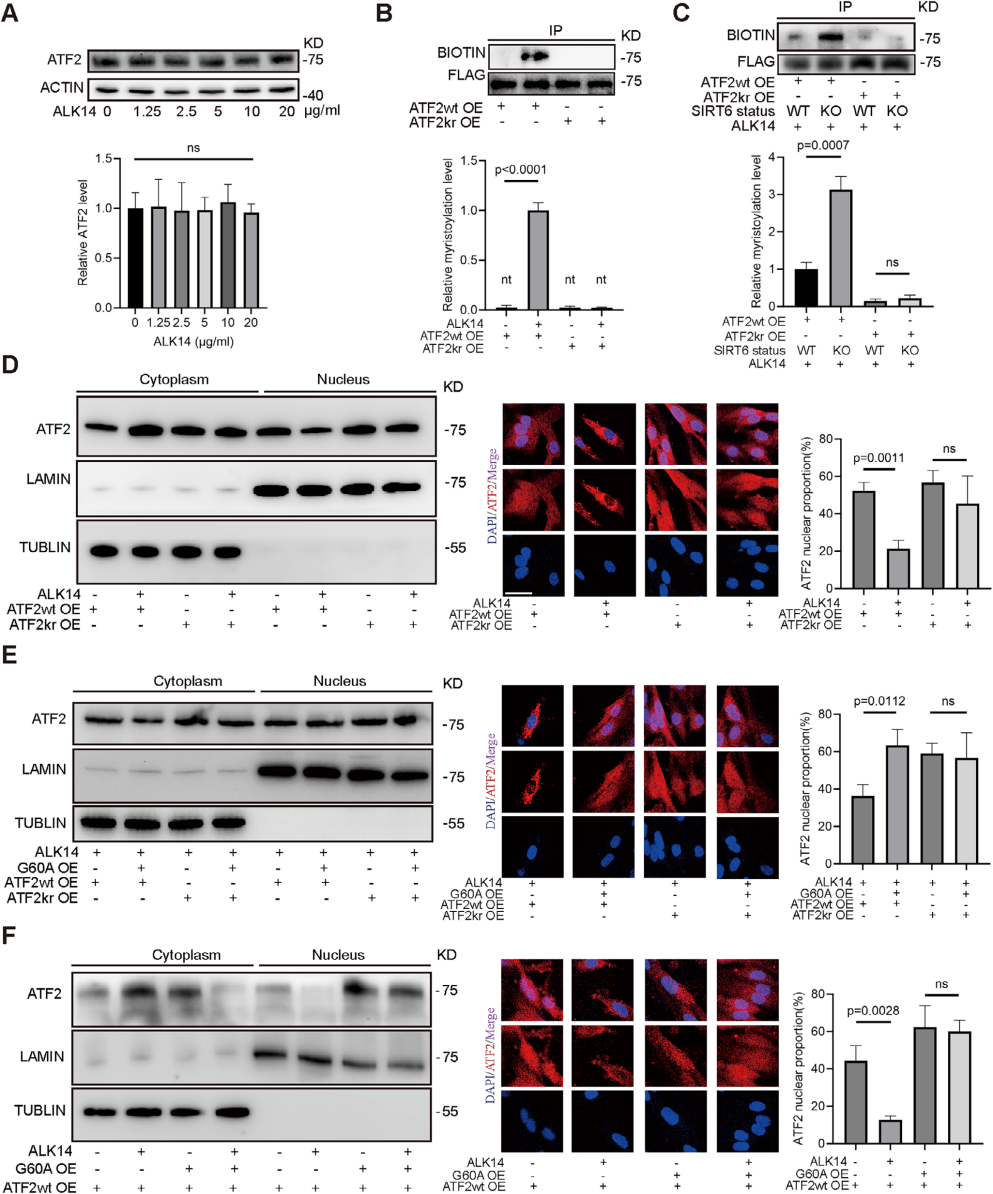
Biological function of myristoylation at K296 site of ATF2. A) Western blot analysis of ATF2 level in 293T under ALK14 treatment in a concentration gradient (0–20 µg mL^−1^) for 12 h (n = 3). B) Immunoprecipitation (IP) and CLICK IT assays were performed in the lysates of SIRT6 KO 293T to detect the myristoylation level of ATF2 (n = 3). Before lysis, cells were transfected with ATF2‐FLAG (WT) and ATF2 K296R‐FLAG (KR) plasmids treated with or without ALK14 (10 µg mL^−1^) for 12 h. C) IP and CLICK IT assays were performed in the lysates of SIRT6KO and WT cells to detect the myrsitoylation of ATF2 (n = 3). Before lysis, cells were transfected with ATF2‐FLAG (WT) and ATF2 K296R‐FLAG (KR) plasmids under ALK14 (10 µg mL^−1^) treatment for 12 h. D) Nucleoplasmic separation and western blot assays were performed in SIRT6 KD HMEC cells to detect the level of ATF2 in cytoplasm and nucleus. Before lysis, cells were transfected with ATF2 WT and KR plasmids under ALK14 (10 µg mL^−1^) treatment for 12 h or not. Immunofluorescence staining of ATF2 (red) in each group under ALK14 treatment or not. DAPI was used for counterstaining cellular nuclei (blue) (n = 10, scale bars = 20 µm). The histograms show the relative ATF2 level (%) in nuclear (n = 3). E) Nucleoplasmic separation and western blot assays were performed in SIRT6 KD HMEC cells to detect the level of ATF2 in cytoplasm and nucleus. Before lysis, cells were transfected with G60A, ATF2 WT and KR plasmids under ALK14 (10 µg mL^−1^) treatment for 12 h. Immunofluorescence staining of ATF2 (red) in each group under ALK14 treatment. DAPI was used for counterstaining cellular nuclei (blue) (n = 10, scale bars = 20 µm). The histograms show the relative ATF2 level (%) in nuclear (n = 3). F) Nucleoplasmic separation and western blot assays were performed in SIRT6 KD HMEC cells to detect the level of ATF2 in cytoplasm and nucleus. Before lysis, cells were transfected with G60A and ATF2 WT plasmids under ALK14 (10 µg mL^−1^) treatment for 12 h or not. Immunofluorescence staining of ATF2 (red) in each group under ALK14 treatment or not. DAPI was used for counterstaining cellular nuclei (blue) (n = 10, scale bars = 20 µm). The histograms show the relative ATF2 level (%) in nuclear (n = 3). Date is represented as means ± S.E.M. *p* value by two‐tailed *t*‐test (A‐F).

We initiated our investigation by assessing ALK14 concentration effects on ATF2 expression in vitro. ATF2 protein levels remained stable across ALK14 concentrations (0–20 µg mL^−1^, 12‐hour treatment) indicating the stability of ATF2 protein levels under ALK14 treatment (**Figure** **3**A). To examine ATF2 myristoylation in vitro, we transfected 293T cells with FLAG‐tagged ATF2 plasmids (Figure S5A–F, Supporting Information).  Following ALK14 treatment and FLAG‐bead immunoprecipitation, we observed significant myristoylation enhancement upon ALK14 exposure, which was further amplified in SIRT6 KO cells, confirming SIRT6‐mediated demyristoylation (Figure 3B,C). Site‐directed mutagenesis (K296R) substantially reduced ATF2 myristoylation compared to wild‐type. Notably, SIRT6 KO no longer enhanced myristoylation in the K296R mutant, confirming K296 as the primary SIRT6‐regulated myristoylation site. Parallel assessment of ATF2 acetylation confirmed SIRT6 does not modulate ATF2 acetylation, underscoring its specific demyristoylase activity (Figures S4C and S5G, Supporting Information).

Given SIRT6's role in modulating ATF2 nuclear accumulation and myristoylation's involvement in protein localization,^[^ ^33^, ^34^ ^]^ we investigated whether SIRT6‐mediated myristoylation at K296 affects ATF2 nuclear localization. After transfecting FLAG‐ATF2 and its K296R mutant into 293T cells, we fractionated cytoplasmic and nuclear components. ALK14 treatment significantly reduced nuclear FLAG‐ATF2 accumulation, an effect attenuated in the mutant group (Figure S6A,B, Supporting Information). Enhanced ATF2 nuclear‐cytoplasmic shuttling was observed in SIRT6 KO versus WT cells, with minimal differences between mutant forms across conditions (Figure S6C,D, Supporting Information). In HUVECs, SIRT6 overexpression mitigated ALK14‐induced translocation of endogenous ATF2 from nucleus to cytoplasm (Figure S6E, Supporting Information). To further confirm SIRT6's demyristoylation‐specific effect in endothelial cells, we examined ATF2 localization in HMECs transfected with G60A plasmids. ALK14 reduced nuclear accumulation of wild‐type ATF2 in endothelial cells, but not the K296R mutant (Figure 3D; Figure S7A, Supporting Information). G60A enhanced nuclear accumulation of wild‐type ATF2 yet minimally affected the mutant (Figure 3E; Figure S7B, Supporting Information). Further, ALK14‐induced nucleocytoplasmic transport alterations was reduced by G60A (Figure 3F; Figure S7C, Supporting Information). These results establish ATF2 as a myristoylated protein whose K296 modification is reversed by SIRT6, directly regulating ATF2 nucleocytoplasmic translocation.

### 2.4 Signal Pathways and Targeted Molecules Regulated by ATF2 Myristoylation

**Figure 4:**
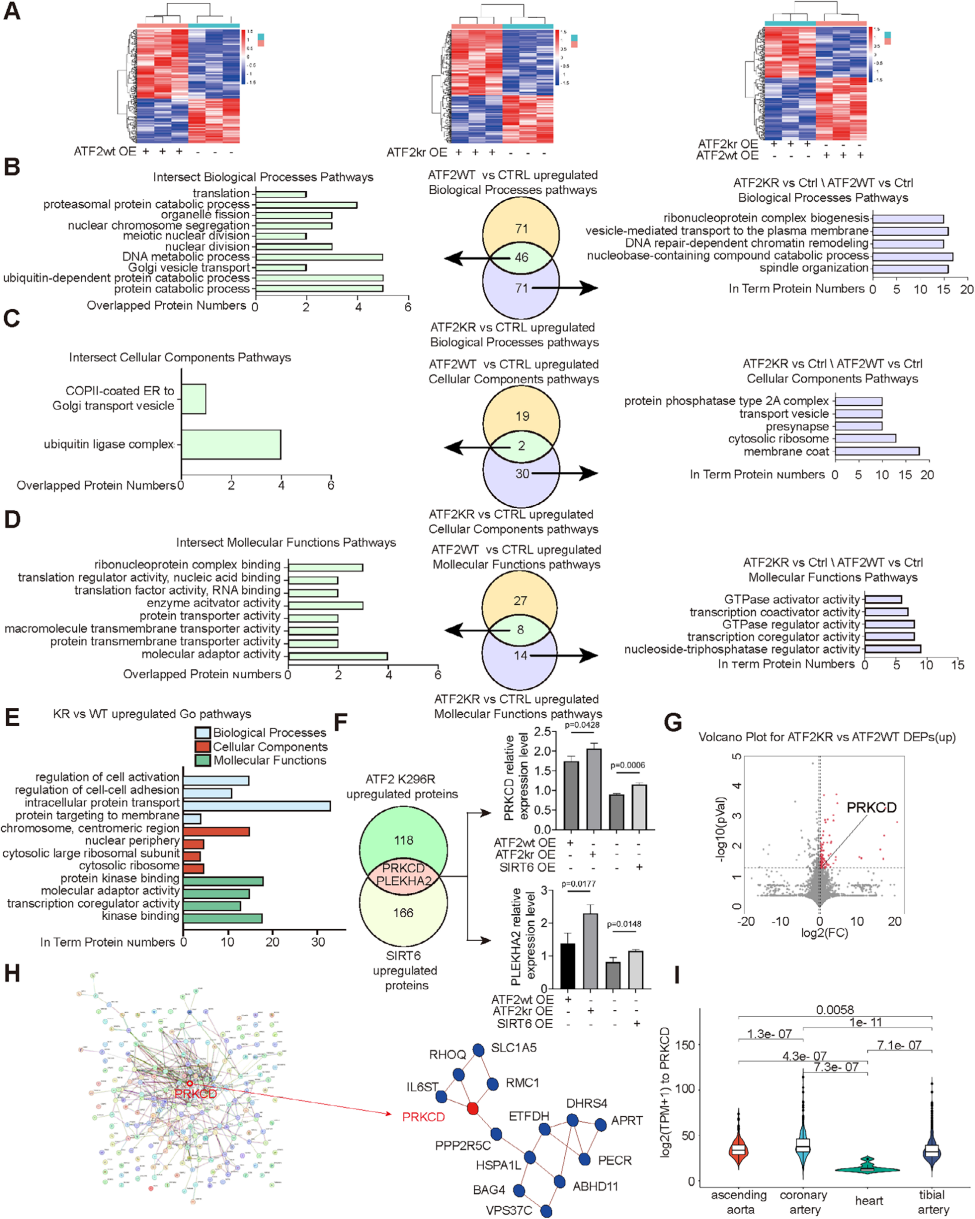
Signal pathways and targeted genes regulated by ATF2 myritoylation. A) The heatmaps of the identified proteins derived from 4D‐Label‐Free proteomics analyses of WT versus Ctrl (left), KR versus Ctrl (middle) and KR versus WT (right) groups (n = 3). Pearson correlation coefficients were performed to measure the distance and average of sample clustering. B–D) The venn's diagrams show the upregulated GO biological processes, cellular components, and molecular functions pathways in WT versus Ctrl (yellow) and KR versus Ctrl (purple). The histograms show GO biological processes, cellular components, and molecular functions pathways in the intersected and KR versus ctrl ∖WT versus Ctrl. The x‐axis shows the number of proteins. E) The histograms showing GO biological processes, cellular components and molecular functions pathways in the upregulated KR versus WT and x‐axis show the number of proteins. F) The venn's diagram shows the overlapped differentially expressed proteins (DEPs) in KR upregulated proteins (KR versus Ctrl, KR versus WT) and SIRT6 upregulated proteins (SIRT6 overexpression versus control). (The data of DEPs in SIRT6 comes from our previous published data^[^ ^35^ ^]^). The histogram shows the relative expression level of PRKCD and PLEKHA2. G) The volcano plots of DEPs derived from KR versus WT groups with PRKCD and PLEKHA2 annotated on the diagram (|log2 (Fold change) |≥0.2, and P <0.05). H) The protein interaction network of the detailed(left) and brief (right) cluster from KR versus WT DEPs, constructed by String (https://cn.string‐db.org/) and metascape (www.metascape.org/). I) The expression of PRKCD in different tissues (heart, tibial artery, coronary artery, ascending aorta) from donors based on data obtained from the GTEx database. The x‐axis shows the tissues from which the 1104 samples were derived, and the y‐axis indicated the log2 (TPM + 1) value.

To demonstrate the signal pathways modulated by ATF2 myristoylation, HMEC‐1 cells were transfected with either ATF2 WT, ATF2 K296R overexpression mutants, or empty vectors, and treated with ALK14 (10 µg mL^−1^) for 12 h, prior to four‐dimensional label‐free MS. Plasmid quantities were standardized across conditions, with triplicate samples ensuring analytical robustness (**Figure** **4**A). Differentially expressed proteins (DEPs) meeting stringent criteria (|log_2_ (Fold Change) | ≥ 0.2, *p* < 0.05) underwent GO interrogation. Analyses of ATF2 WT versus CTRL and mutant versus CTRL groups consistently highlighted translational regulation pathways—including ribosomal complex binding, RNA‐binding translation factors, and nucleic acid‐associated translation regulators—demonstrating preserved transcriptional competence at K296. In contrast, mutant‐exclusive pathways featured membrane‐proximal processes: COPII vesicle coat assembly, transport vesicle dynamics, plasma membrane‐directed trafficking, and GTPase activator functions (Figure 4B–D). Direct WT‐mutant comparisons revealed mutation‐dependent enrichment in cellular adhesion mechanisms, intracellular protein shuttling, and membrane‐targeting pathways, consistent with ATF2 myristoylation governing subcellular localization (Figure 4E). Bioinformatic screening for Myr‐ATF2 K296 targets cross‐referenced upregulated DEPs in mutant cohorts (versus WT/CTRL) with our prior SIRT6‐overexpression dataset.^[^ ^35^ ^]^ Protein kinase C‐delta (PRKCD) and PLEKHA2 emerged as intersecting proteins (Figure 4F).  Integrative volcano plot and protein‐protein interaction (PPI) network analyses demonstrated PRKCD as a central effector (Figure 4G,H). GTEx database interrogation revealed pronounced PRKCD expression in vasculature versus cardiac tissue, underscoring endothelial relevance (Figure 4I). Collectively, ATF2 myristoylation orchestrates membrane‐trafficking paradigms, wherein Myr‐ATF2 (K296)‐targeted PRKCD likely stabilizes endothelial linker proteins via GTPase phosphorylation cascades,^[^ ^36^, ^37^ ^]^ thereby modulating essential vascular behaviors.

### 2.5 SIRT6 Demyristoylates ATF2 to Enhance Endothelial Barrier Function Through the ATF2/PRKCD/VE‐Cadherin Signaling Pathway

**Figure 5:**
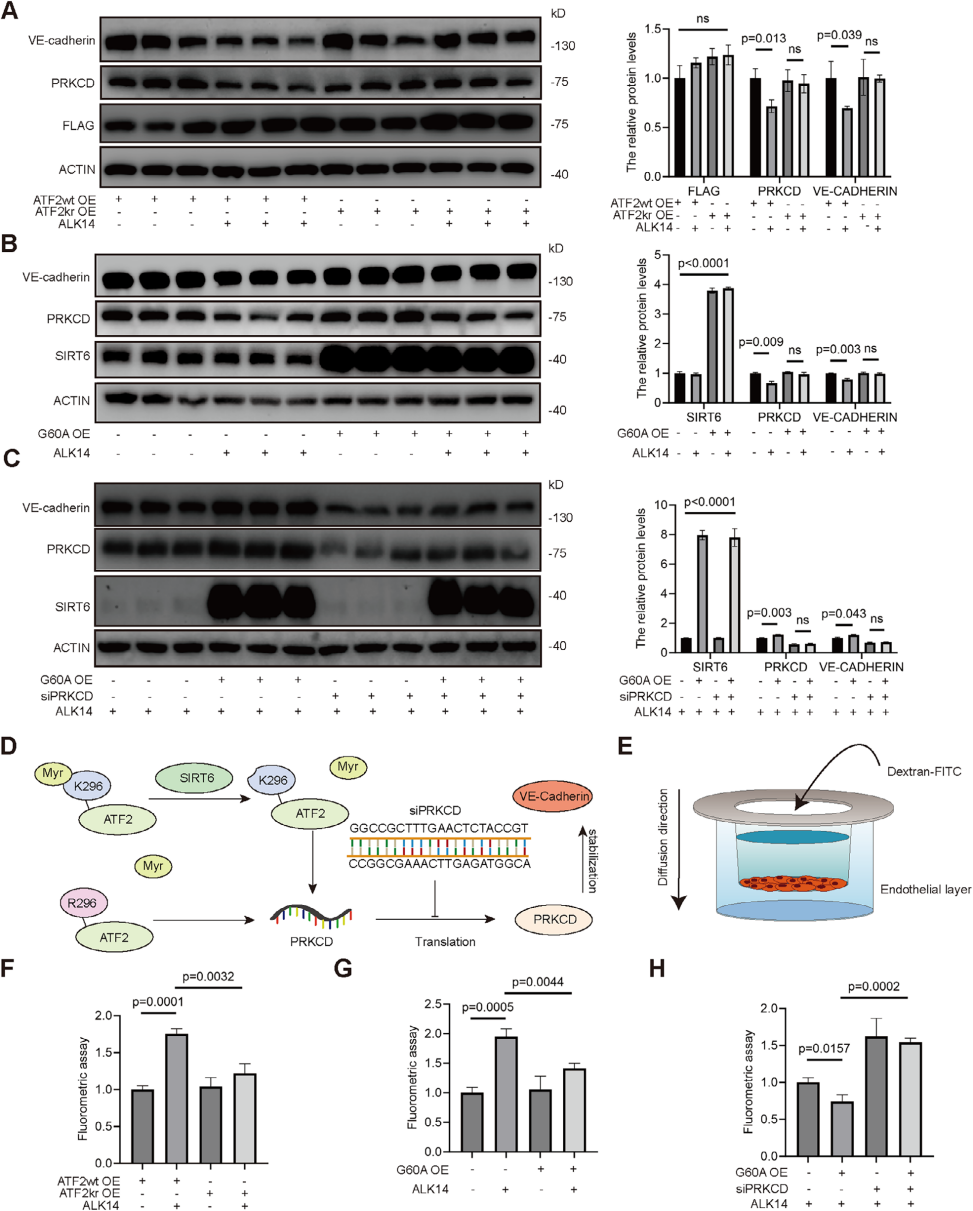
SIRT6 demyristoylated ATF2 to improve the endothelial permeability through PRKCD/VE‐Cadherin signaling pathway. A) Western blot analysis of VE‐Cadherin, FLAG, PRKCD level in HMEC‐1 transfected with ATF2 WT and KR plasmids treated with or without ALK14 (10 ug mL^−1^) for 12h. The histograms show the relative protein level (%) (n = 3). (B) Western blot analysis of VE‐Cadherin, PRKCD, SIRT6 level in HMEC‐1 transfected with G60A plasmids with or without ALK14 treatment. The histograms show the relative protein level (%) (n = 3). C) Western blot analysis of VE‐Cadherin, PRKCD, SIRT6 level in HMEC‐1 transfected with siPRKCD and G60A with ALK14 treatment. The histograms show the relative protein level (%) (n = 3). D) A brief schematic diagram of the role of siPRKCD in ATF2/PRKCD/VE‐Cadherin signaling pathway. E) A schematic diagram of the transwell permeability model. F) The histograms represented the quantitative assays of endothelial permeability in ATF2 WT and KR HMEC‐1 with or without ALK14 treatment (n = 3). G) The histograms represented the quantitative assay of endothelial permeability in HMEC‐1 transfected with G60A plasmids under ALK14 treatment (n = 3) or not. H) The histogram represented the quantitative assay of endothelial permeability of HMEC‐1 transfected with siPRKCD and G60A under ALK14 treatment (n = 3). Date is represented as means ± S.E.M. *p* value by two‐tailed *t*‐test (A, B, C, E, F, G, H).

Endothelial cell function is crucial for maintaining vascular permeability. Impairment of the endothelial barrier increases vascular permeability, with abnormal expression of vascular endothelial‐cadherin (VE‐Cadherin) playing a pivotal role in regulating this microvascular permeability. To elucidate the role of SIRT6 and ATF2 myristoylation in endothelial barrier regulation, HMEC‐1 cells treated with ALK14 exhibited significantly reduced VE‐cadherin expression (**Figure** **5**A; Figure S8A, Supporting Information), indicating compromised barrier function from excessive myristoylation. Concomitant PRKCD reduction was rescued by SIRT6 overexpression or ATF2 K296R mutation (Figure S8A,B, Supporting Information). SIRT6‐mediated VE‐cadherin upregulation required intact PRKCD expression (Figure S8C, Supporting Information), while expression of deacetylase‐defective SIRT6 G60A prevented ALK14‐induced PRKCD and barrier impairment (Figure 5B). G60A overexpression also reversed VE‐cadherin suppression following PRKCD knockdown (Figure 5C). confirming that SIRT6 demyristoylates ATF2‐K296 to augment PRKCD/VE‐cadherin expression under ALK14 stimulation (Figure 5D). Transwell assays using fluorescent dextran revealed ALK14‐induced permeability in ATF2 WT cells, which was absent in K296R mutants regardless of treatment (Figure 5E,F; Figure S8D, Supporting Information). Both SIRT6 OE and G60A OE blocked permeability increases (Figure 5G; Figure S8E, Supporting Information), with significantly lower flux than WT under ALK14 stimulation. PRKCD knockdown abolished this protection (Figure 5H; Figure S8F, Supporting Information). Neither ALK14 nor ATF2 modification influenced endothelial‐stromal differentiation (Figure S8G, Supporting Information).

**Figure 6:**
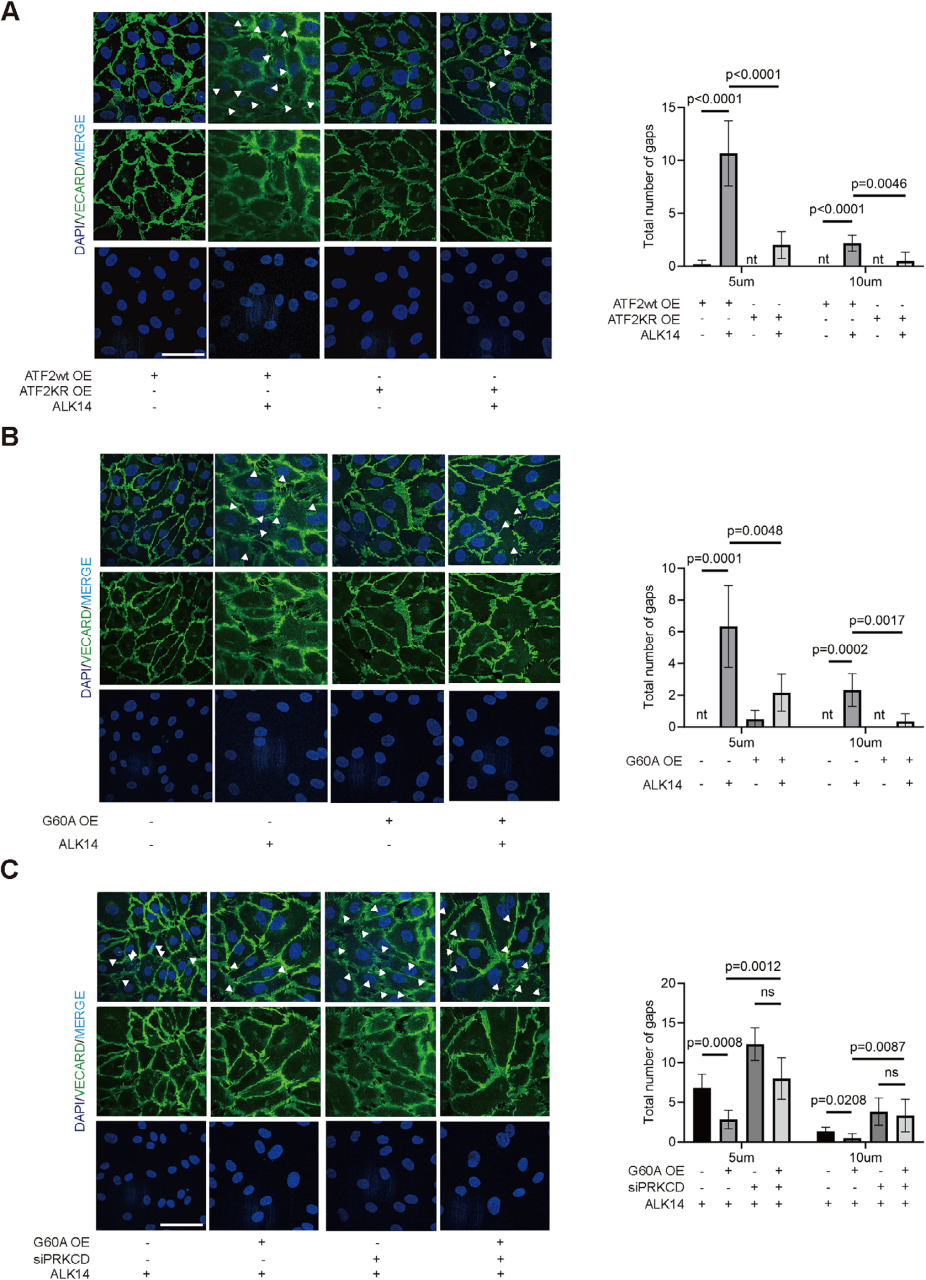
SIRT6 demyristoylated ATF2 to improve the endothelial permeability through PRKCD/VE‐Cadherin signaling pathway. A) Immunofluorescence staining analyses of VE‐Cadherin (green) in HMEC‐1 transfected with ATF2 WT and KR plasmids under ALK14 (10 µg mL^−1^) treatment for 12 h or not. Nuclei were counterstained with DAPI (blue) (n = 8–10, scale bars = 50 µm). The histograms show the distribution of the intercellular 5um or 10um gaps of each group (n = 8–10). B) Immunofluorescence staining analyses of VE‐Cadherin (green) in HMEC‐1 transfected with G60A plasmids under ALK14 treatment (n = 8–10, scale bars = 50 µm) or not. The histograms show the distribution of intercellular 5um or 10um gaps of each group (n = 8–10). C) Immunofluorescence staining analyses of VE‐Cadherin (green) in HMEC‐1 transfected with siPRKCD and G60A under ALK14 treatment (n = 8–10, scale bars = 50 µm). The histograms show the distribution of the intercellular 5 um or 10um gaps of each group (n = 8–10). Data were presented as mean ± S.E.M and analyzed using a two‐tailed *t*‐test A,B,C). ns indicates no significant difference.

Confocal microscopy confirmed ALK14‐induced gap formation (5 µm/10 µm) in ATF2 WT endothelia, prevented by K296R mutation (**Figure** **6**A; Figure S9A, Supporting Information). While SIRT6/G60A OE reduced gaps in ATF2 WT cells (Figure 6B; Figure S9B, Supporting Information), this effect required intact PRKCD expression (Figure 6C; Figure S9C, Supporting Information). Control experiments with natural myristic acid versus ALK14 in HUVECs showed equivalent impacts on ATF2 localization and the PRKCD/VE‐cadherin axis, excluding structural differences between ALK14 and natural coumarin acid (Figure S10A–F, Supporting Information). Mechanistically, luciferase/ChIP‐qPCR/CO‐IP assays demonstrated ATF2 and SIRT6‐G60A enhance PRKCD transcription independently of ALK14 without altering its post‐translational modifications (Figure S11A–E, Supporting Information). Collectively, SIRT6 preserves endothelial integrity not through deacetylase activity, but via ATF2‐K296 demyristoylation, driving PRKCD/VE‐cadherin expression to suppress permeability.

### 2.6 Comparative Functional Studies between SIRT6 Mutant and SIRT6 WT

**Figure 7:**
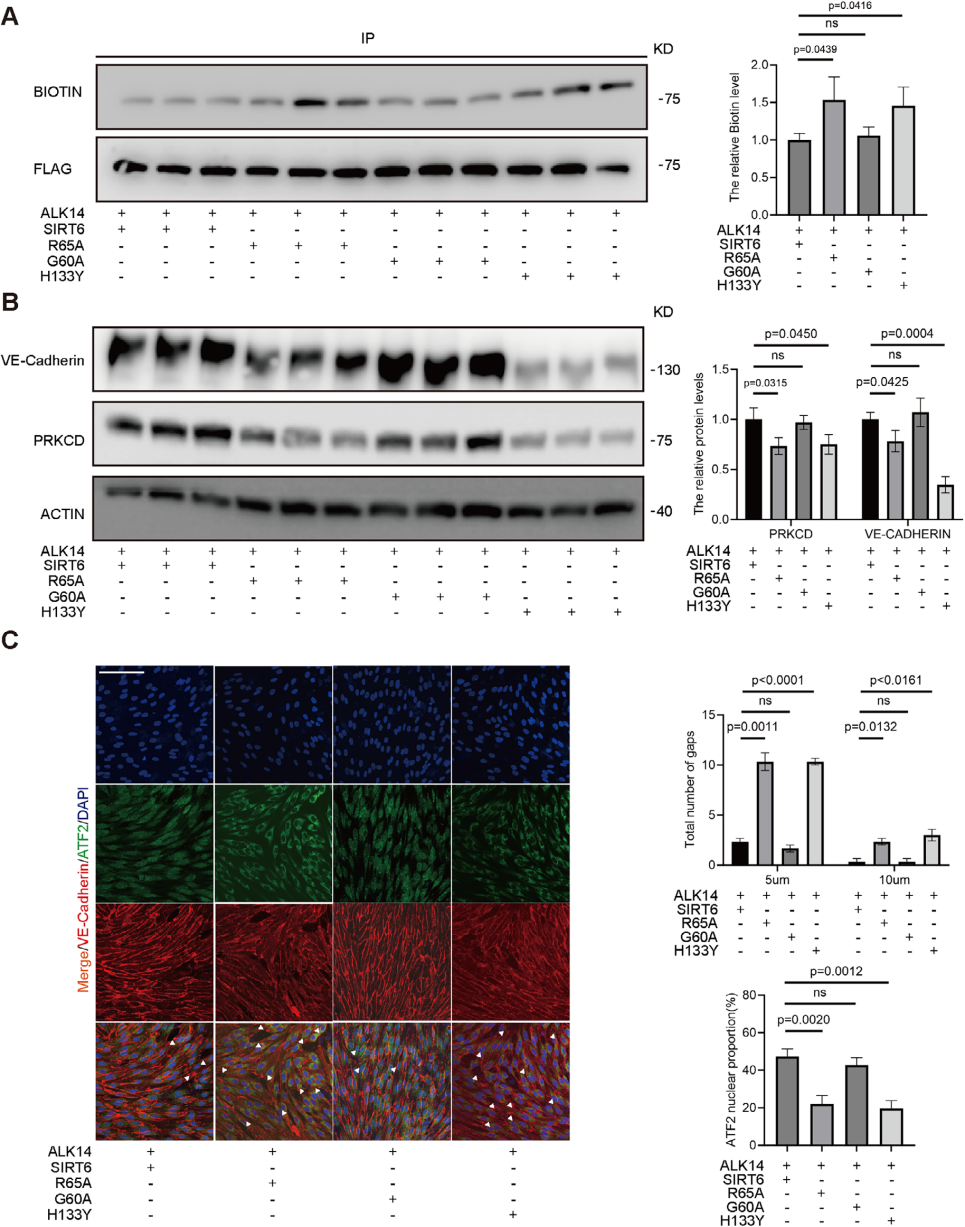
ATF2/PRKCD/VE‐Cadherin signaling pathway was regulated by SIRT6 demyristoylase activity. A) Immunoprecipitation (IP) and CLICK IT assays were performed in the lysates of 293T SIRT6KO cells to detect the myristoylation level of ATF2 (n = 3). Before lysis, cells were transfected with SIRT6 mutants plasmids treated with ALK14 (10 µg mL^−1^) for 12 h. The histograms show the relative ATF2 myristoylation level (%) (n = 3). B) Western blot analysis of VE‐Cadherin, PRKCD level in HUVECs transfected with SIRT6 and its mutants plasmids treated with ALK14 (10 ug mL^−1^) for 12h. The histograms show the relative protein level (%) (n = 3). C) Immunofluorescence staining analyses of VE‐Cadherin (red) and ATF2(green) in HUVECs transfected with SIRT6 and its mutants treated with ALK14 (10 µg mL^−1^) for 12 h. The histograms (up) show the distribution of the intercellular 5 or 10 um gaps of each group. The histograms (down) show the ATF2 nuclear proportion. scar bar = 100 µm (n = 6–8) Data were presented as mean ± S.E.M and analyzed using a two‐tailed *t*‐test. ns indicates no significant difference.

To functionally dissect SIRT6 activities, parallel experiments were performed comparing: SIRT6 WT (competent in both deacetylase and demyristoylase activities), the R65A mutant (exclusively deficient in demyristoylase activity), the G60A mutant (exclusively deficient in deacetylase activity), and the catalytic‐dead H133Y mutant. Endogenous ATF2 was immunopurified from cells expressing these SIRT6 variants and assessed for myristoylation levels using Click‐iT assays. SIRT6 constructs lacking demyristoylase activity exhibited significantly reduced ATF2 demyristoylation capacity (**Figure** **7**A). Western blot analysis revealed that only demyristoylase‐competent SIRT6 variants rescued VE‐cadherin and PRKCD expression (Figure 7B). Complementarily, confocal immunofluorescence demonstrated that the demyristoylation‐active G60A mutant significantly outperformed catalytic‐null controls in maintaining ATF2 nuclear retention and endothelial junction integrity during ALK14 challenge (Figure 7C). These integrated analyses establish that SIRT6 demyristoylase activity—functionally independent of its deacetylase function—specifically protects endothelial homeostasis under ALK14 treatment.

### 2.7 SIRT6 Demyristoylation of ATF2 Stabilizes the Endothelial Barrier In Vivo

**Figure 8:**
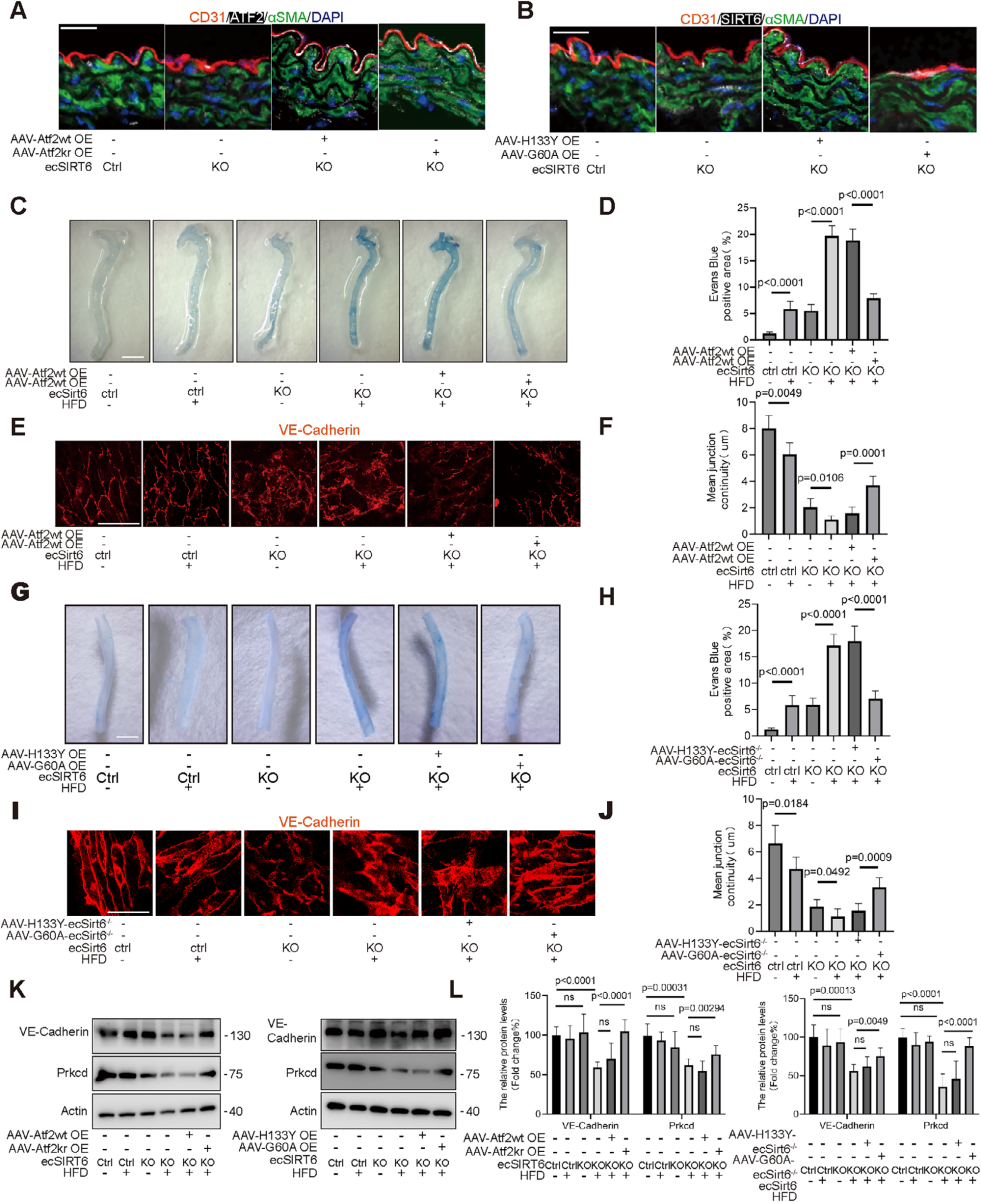
SIRT6 demyristoylated ATF2 to stabilize endothelial cells barrier in vivo. A) Immunofluorescence staining analyses of CD31 (red) αSMA (green) and ATF2 (white) in the aortic section from mice post AAV injection. Nuclei were counterstained with DAPI (blue) (n = 6–8, scale bars = 30 µm). B) Immunofluorescence staining analyses of CD31 (red) αSMA (green) and SIRT6 (white) in the aortic section from mice post AAV injection. Nuclei were counterstained with DAPI (blue) (n = 6–8, scale bars = 30 µm). C) The permeation of the Evans blue dye into the descending aortas of each group. (D) The histograms showed the quantification of Evans blue dye in the aortas, scar bar = 2.5 mm (n = 6). E) *En face* immunofluorescence staining images of VE‐Cadherin in the descending aortas of each group. F) The histogram showed the quantification of VE‐Cadherin mean junctions continuity in the aortas, scar bar = 25 µm (n = 6). G) The permeation of the Evans blue dye into the descending aortas of each group. H) The histograms showed the quantification of Evans blue dye in the aortas, scar bar = 2.5 mm (n = 6). I) En face immunofluorescence staining images of VE‐Cadherin in the descending aortas of each group. J) The histogram showed the quantification of VE‐Cadherin mean junctions continuity in the aortas, scar bar = 25 µm (n = 6). K) Western blot analyses of VE‐Cadherin, Prkcd expression in each group (n = 6). L)The histogram showed the relative ATF2, SIRT6 expression (n = 6). Date is represented as means ± S.E.M. *p* value by two‐tailed *t*‐test (C‐L).

To investigate the in vivo effects of SIRT6 and ATF2 myristoylation on endothelial barrier function, we generated endothelial‐specific SIRT6 KO mice, subsequently creating SIRT6 KO‐ATF2 WT OE and SIRT6 KO‐ATF2 K298R mutant OE models via adeno‐associated virus (AAV)‐mediated delivery (Figure S11F, Supporting Information). High‐fat diet feeding was used to accelerate myristoylation modification in vivo, leading to endothelial barrier dysfunction, Successful high‐fat diet (HFD) modeling was confirmed by blood lipid alterations (Figure S11G, Supporting Information). Immunofluorescence, RT‐qPCR, and western blot analyses validated efficient SIRT6 KO and ATF2 overexpression in mouse aortic endothelial cells (MAECs) (**Figure** **8**,B; Figure S11H,I, Supporting Information). Evans blue permeability assays revealed increased vascular leakage in SIRT6 KO mice, exacerbated by HFD, whereas SIRT6 KO mice expressing the ATF2 K298R mutant exhibited significantly reduced permeability (Figure 8C,D). EN FACE evaluations confirmed that endothelial barrier impairment from SIRT6 KO and HFD was substantially rescued by ATF2 K298R overexpression, indicating that SIRT6 deficiency compromises barrier function through deficient ATF2‐K296 demyristoylation (Figure 8E,F). Specificity testing via AAV‐mediated delivery of catalytic mutants into ecSIRT6^−/−^ mice demonstrated that G60A overexpression—but not H133Y overexpression—alleviated permeability increases (Figure 8G. and H) and restored barrier integrity in EN FACE assessments (Figure 8I,J). Western blot analysis of primary aortic endothelia further showed that both ATF2 K298R and SIRT6 G60A rescued HFD‐ and SIRT6 KO‐induced reductions in PRKCD and VE‐cadherin expression (Figure 8K,L; Figure S12A,B, Supporting Information).

### 2.8 In Vivo Validation of the SIRT6/ATF2/PRKCD Axis and Clinical Connection and Transformation

**Figure 9:**
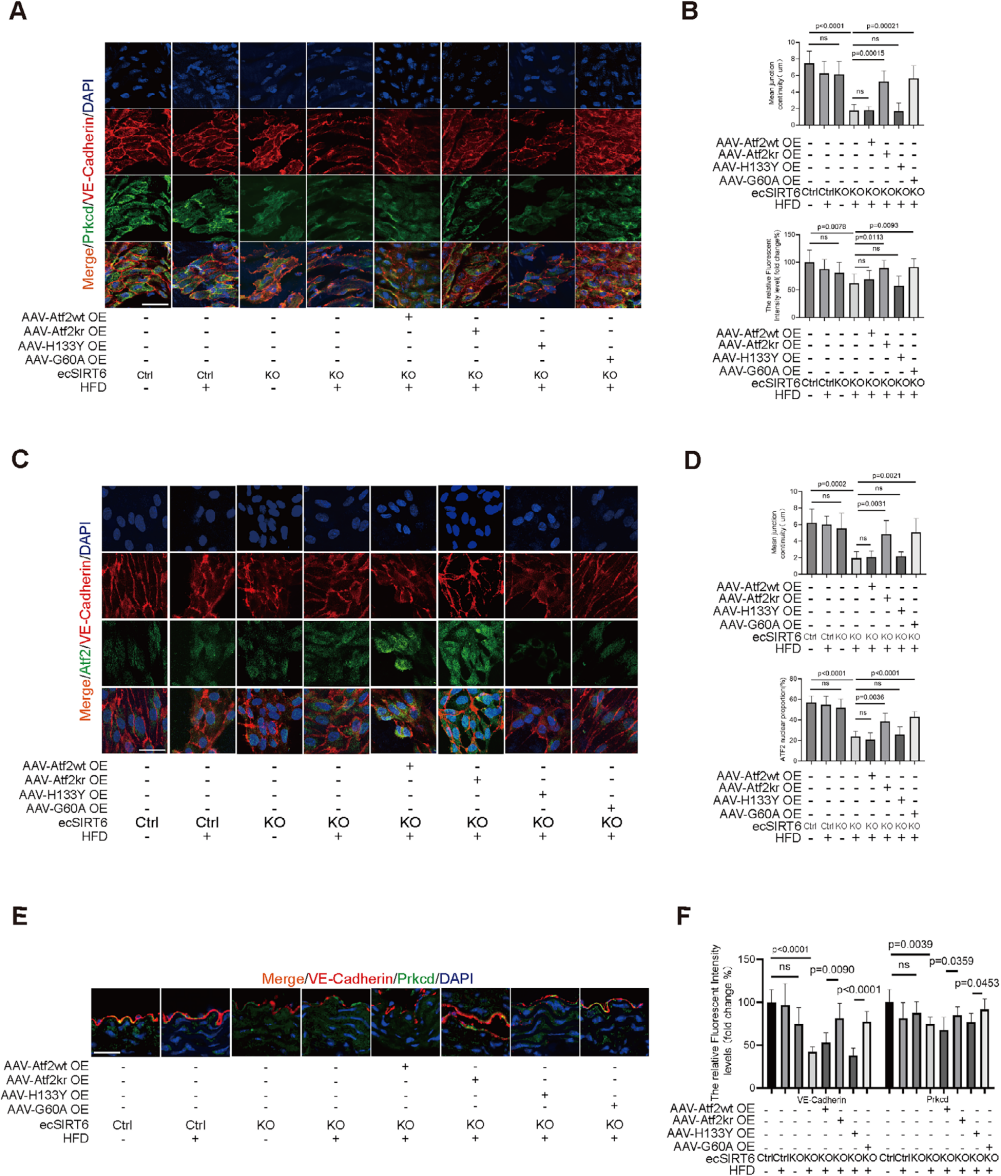
In vivo validation of the SIRT6/ATF2/PRKCD axis A) En face immunofluorescence staining images of Prkcd (green), VE‐Cadherin (red) in the descending aortas of each group. Nuclei were counterstained with DAPI (blue). B) The histogram (up) showed the quantification of VE‐Cadherin mean junctions continuity in the aortas. The histogram (down) showed the relative fluorescent intensity (fold change%) of Prkcd in the aortas, scar bar = 25 µm (n = 6–8). C) Immunofluorescence staining analyses of Atf2(green) and VE‐Cadherin (red) in primary aortic endothelial cells. Nuclei were counterstained with DAPI (blue). D) The histogram (up) showed the quantification of VE‐Cadherin mean junctions continuity in the aortas. The histogram (down) showed the Atf2 nuclear proportion in the aortas, scar bar = 15 µm (n = 6–8). E) Immunofluorescence staining analyses of VE‐Cadherin (red) and Prkcd (green) on sections of mouse descending aorta. DAPI was used for counterstaining cellular nuclei (blue). F) The histogram showed the relative fluorescent intensity levels of VE‐Cadherin and Prkcd in the aortas. scar bar = 30 µm (n = 6–8). Data were presented as mean ± S.E.M and analyzed using a two‐tailed *t*‐test. ns indicates no significant difference.

EN FACE immunofluorescence demonstrated that both ATF2 K298R overexpression and SIRT6‐G60A (retaining demyristoylase activity) enhanced aortic endothelial junction integrity and increased PRKCD fluorescence intensity in vivo (**Figure** **9**A,B; Figure S13A, Supporting Information). Immunofluorescence of primary aortic endothelia further revealed that either the ATF2 mutant or SIRT6‐G60A rescued the HFD‐induced reduction in nuclear ATF2 localization under SIRT6‐deficient conditions (Figure 9C,D). Aortic section fluorescence confirmed endothelial‐specific restoration of HFD/SIRT6 KO‐impaired PRKCD expression by ATF2 K298R or G60A, with no effect observed in fibroblasts (Figure 9E,F).

Further, fluorescence imaging in muscle tissue reveals that endothelial‐specific SIRT6 knockout induces systemic vascular permeability dysregulation (Figure S13B, Supporting Information). Concurrently, *en face* analysis demonstrates that reduced VCAM‐1 expression—resulting from high‐fat diet and SIRT6 deletion—is rescued by the G60A mutant, demonstrate SIRT6's multifaceted regulation of endothelial function (Figure S13C, Supporting Information).

**Figure 10:**
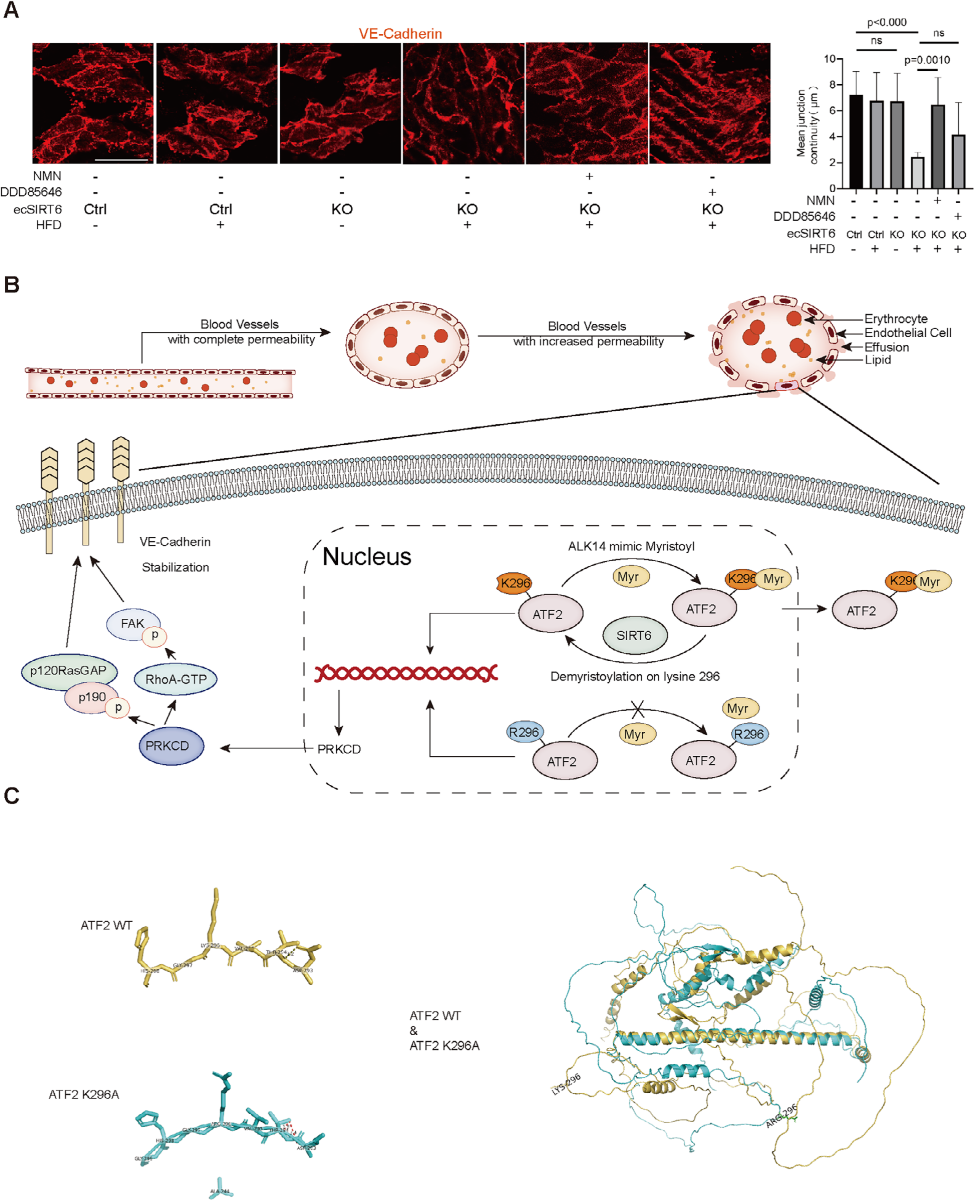
Clinical transformation (A) En face immunofluorescence staining images of VE‐Cadherin in the descending aortas of each group. NMN (100 mg kg^−1^ day^−1^) and DDD85646 (100 mg kg^−1^ day^−1^). The histogram showed the quantification of VE‐Cadherin mean junctions continuity in the aortas, scar bar = 25 µm(n = 6–8). B) The schematic representation for the role of SIRT6 in maintaining endothelial barrier via ATF2 K296/PRKCD/VE‐Cadherin signaling. C) Structure of Wild‐Type ATF2 and K296R ATF2. Top: detailed structure of 296K and 8Å around; Middle: detail structure of 296R and 8Å around; Down: Overall structural comparison of wild‐type ATF2 and K296R ATF2 predicted by AlphaFold2. Date is represented as means ± S.E.M. *p* value by two‐tailed *t*‐test (A).

Notably, transcriptomic analysis of human aortic data (GSE100927) revealed significant SIRT6 downregulation in atherosclerotic versus control samples across arterial beds, indicating the potential role of SIRT6 in vascular diseases (Figure S13D, Supporting Information). Therapeutic administration of NMN (100 mg kg^−1^ day^−1^) demonstrated that NMN restored endothelial integrity in *en face* assessments, suggesting that other SIRT family members with demyristoylase activity may coordinately contribute to endothelial barrier protection (**Figure** **10**A). These in vivo findings align with mechanistic in vitro data (Figure 10B), establishing that endothelial barrier regulation critically relies on SIRT6‐mediated ATF2‐K296 demyristoylation, emphasizing the pivotal role of the ATF2/PRKCD/VE‐cadherin signaling axis in maintaining vascular integrity.

## 3 Discussion

Epigenetic modifications—including DNA methylation and post‐translational modifications (PTMs) such as phosphorylation, acetylation, methylation, and ubiquitination—play pivotal roles in regulating genome function, thereby influencing development and biological behavior. Among PTMs, protein fatty‐acylation has emerged as a significant regulatory mechanism.^[^ ^38^, ^39^ ^]^ Myristoylation, a long‐chain fatty acylation subtype, critically regulates protein transmembrane transport.^[^ ^33^ ^]^ However, the known lysine‐myristoylated proteins remains limited due to incomplete characterization of defatty‐acylation enzymes and inadequate detection methodologies.^[^ ^40^ ^]^ This study introduces a novel enrichment strategy employing the SIRT6 H133Y mutant (“dSIRT6”), which exhibits high‐affinity myristoyl binding without demyristoylase activity. This approach identified 15 previously unknown human myristoylated proteins in cellular systems. Significantly, we demonstrate that SIRT6 demyristoylates ATF2 at K296, modulating its nucleocytoplasmic transport. This process enhances VE‐Cadherin expression through PRKCD‐dependent signaling, ultimately stabilizing endothelial barrier function.

To optimize myristoyl‐modified peptide enrichment, Mg^2^⁺ was incorporated to enhance H133Y affinity for H3K9‐myr in BLI assays. Although its efficacy in immunoprecipitation (IP) assays remained limited, likely due to H133Y instability, final enrichment was achieved through MyrIP and elution buffer optimization. This approach outperformed conventional methods, including acyl‐specific antibodies and isotope labeling, which face specificity and scalability limitations. Notably, ATF2, a regulator of diverse cellular processes,^[^ ^28^, ^41^ ^]^ was identified as myristoylated at K296, a novel finding that distinguishes it from known ATF2 PTMs. The findings in this study underscore the potential regulatory roles of myristoylation on protein translocation and secretion, which was similar to mechanisms previously described for TNFα.^[^ ^18^ ^]^ Strikingly, the myristoylation site (K296) of ATF2 was not localized in the reported two canonical nuclear localization sequences (NLS) or one export sequence (NES), which was chromosome region maintenance 1(CRM1)‐dependent.^[^ ^42^ ^]^ Based on online published data, we found that the incidence of the ordinary population with the 296 lysine to arginine mutation in ATF2 was about 1/300 000 (https://gnomad.broadinstitute.org/). Further, to investigate the potential biological impact of the mutation of ATF2, AlphaFold was employed to predict the ATF2 structure before and after the mutation, with the result indicating that compared with the wild type, the mutant had a new hydrogen bond formation between 293 and 294, and the overall structure of the two remained relatively unchanged (Figure 10C). For a mutation in a network of hydrogen bonds would affect a stabilizing interaction within a folded protein, the native state may be destabilized, which might be accompanied by loss of function.^[^ ^43^ ^]^ Deep investigation into the relationship between K296 myristoylation and ATF2's traditional localization sequences is needed to elucidate their interaction dynamics. Exploring the downstream molecular mechanisms regulated by SIRT6‐mediated demyristoylation of ATF2, this study employed 4D‐Label‐free proteomics, revealing significant involvement in pathways related to membrane transport and protein activation, which indicated that both might participate in the maintenance of cell membrane structure homeostasis.^[^ ^44^ ^]^ Further analysis was performed by intersecting the overlapped GO pathways of ATF2 mutant group versus ATF2 WT group and SIRT6 OE group versus SIRT6 OENC group,^[^ ^35^ ^]^ and target molecules PRKCD and PLEKH2A were identified, with PRKCD's role in endothelial barrier function^[^ ^36^, ^37^ ^]^ further confirmed by western blot assays, suggesting the SIRT6/ATF2/PRKCD/VE‐Cadherin axis as a protective mechanism for endothelial integrity.

We analyzed available human aortic transcriptomic data on GEO (GSE 10 092 7^[^ ^45^ ^]^), Transcriptomic analysis revealed significant differential expression of SIRT6 between atherosclerotic and control groups across three distinct arterial beds (Figure S13D, Supporting Information). Regarding the observed transcriptional upregulation of SIRT6 in atherosclerotic lesions, this may represent a compensatory response wherein SIRT6 expression is enhanced at the transcriptional level under pathological stress to mediate protective functions.^[^ ^46^ ^]^ Another study found that SIRT6 was markedly downregulated in the radial artery tissue of patients with Vascular calcification.^[^ ^47^ ^]^ Besides, researches determined that the mRNA expression of SIRT6 was the only significantly downregulated sirtuin protein family members at different calcification levels. Further, according to a published Mendelian randomization study,^[^ ^48^ ^]^ GWAS and Mendelian randomization analyses establishes 1‐myristoyl‐2‐arachidonoyl‐GPC (LysoPC (14:0/20:4)) as a causal mediator of peripheral arteriosclerosis. 1‐myristoyl‐2‐arachidonoyl‐GPC is a glycerophospholipid in which a phosphorylcholine moiety occupies a glycerol substitution site.^[^ ^49^ ^]^ The myristic acid moiety assits it to attach to cell membranes. In another research, researchers found that the impairment of endothelial SIRT6 expression links diabetes to HFpEF through the alteration of FA transport across the endothelial barrier.^[^ ^11^ ^]^ These studies on SIRT6 and myrisic acid precursor suggest that SIRT6's role in CVDs may involve complex lipid metabolism interactions—a key direction for future clinical data collection.

SIRT6 has already been proven to protect the endothelial barrier after ischemia‐reperfusion to improve cardiac function through deacetylation in vivo.^[^ ^50^ ^]^ Our in vivo studies using AAV‐mediated ATF2 WT OE and mutant (K296R) OE in endothelial‐specific SIRT6 KO mice corroborated in vitro findings, with the mutant OE group partially improving aortic endothelial barrier disruption induced by a high‐fat diet. Despite challenges in detecting lysine myristoylated proteins in vivo,^[^ ^40^ ^]^ the results from animal models reinforced the potential of targeting SIRT6‐mediated protein myristoylation for therapeutic intervention in vascular permeability‐related diseases. Previous research has showen that SIRT6(±); ApoE(‐/‐) mice after HFD feeding exhibited exacerbated atherosclerotic lesion development, concurrent with increased expression of the proinflammatory cytokine VCAM‐1.^[^ ^51^, ^52^ ^]^ We also conducted additional tests for inflammation markers VCAM‐1 in the endothelium by enface staining. The results showed that G60A overexpression reduced the level of VCAM‐1 caused by SIRT6 deficient and HFD, indicating that the demyristoylase activity of SIRT6 on endothelial function has multiple protective functions.

In this study, ATF2 was verified to be demyristoylated by SIRT6 at K296 to regulate endothelial barrier function in vitro and in vivo, which indicated that SIRT6 might be a potential intervention drug target for the protein myristoylation modulation and restoring the conversion balance between blood and interstitial fluid. Currently, some SIRT6 activators (**Table** **1**) and inhibitors (**Table** **2**) with moderate EC50s were reported; nevertheless, most of them were only investigated on the deacetylase activity of SIRT6. We employed NMN, a broad‐spectrum activator of sirtuin (SIRT) family members, and NMT, the catalytic enzyme for N‐terminal myrisotylation to evaluate their therapeutic effect in mice model. The results demonstrate that NMN partially rescued endothelial damage induced by high‐fat diet in SIRT6‐deficient endothelium, potentially attributable to its broad‐spectrum enhancer activity on other SIRT members with demristoylase activity like SIRT2,^[^ ^53^ ^]^ thereby compensating for the loss of SIRT6‐mediated protective mechanisms. The suboptimal therapeutic efficacy of DDD85646 (100 mg kg^−1^ day^−1^) likely stems from the reported sequence specificity governing N‐myristoyltransferase (NMT) substrate recognition. NMT selectively catalyzes myristoylation at lysine residues with S6 K7 sequence.^[^ ^54^ ^]^ The K296 residue of ATF2 is located distally from the N‐terminal region and thus may not be an efficient substrate for NMT‐mediated myristoylation. Accordingly, as a subsequent step, the discovery of new SIRT6‐targeted drugs that moderate the protein myristoylation and vascular permeability would be carried out urgently. Our study highlighted SIRT6's role in protecting endothelial barrier function via the ATF2 K296^Myr^/PRKCD/VE‐Cadherin pathway. These insights pave the way for developing novel therapeutic strategies against vascular permeability and related diseases, emphasizing the significance of SIRT6 as a drug target for modulating protein myristoylation and vascular health.

**Table 1:** The activators of SIRT6 were represented with structure, EC_50_, category, company, indications, and reference.

Activator	EC_50_	Category	Company	Indication	Refs.
MDL‐800	90.4 µm	Selective activator	Medicinal Bioinformatics Center, Shanghai JiaoTong University, zhang jian	Reduce cardiac lipid accumulation	[57]
MDL‐801	5.7 µm	Selective activator		/	[57]
UBCS039	38 µm	Selective activator	Universität Bayreuth	Trigger lethal autophagy	[58]
Rosiglitazone	/	Marketable drugs	–	Ameliorate hepatic steatosis	[59]
Chrysophanol	/	Natural extract	–	Diminish inflammatory reaction of OA	[60]
Cyanidin	/	Natural extract	–	Ameliorate the progression of osteoarthritis	[61]
Egtc	/	Natural extract	–	Protecte against endothelial senescence	[62]
Fluvastin	/	Marketable drugs	–	Cholesterol regulation	[63]

**Table 2:** The inhibitors of SIRT6 were represented with structure, IC_50_, category, company, indications, and reference.

Inhibitor	IC_50_	Category	Company	Indication	Refs.
Diquercetin	130 µm	Natural extract	Constructed by Vladimír Heger	/	[64]
5‐(4‐methylpiperazin‐1‐yl)‐2‐nitroaniline	4.93 µm	Selective inhibitor	Constructed by Weining Sun	Reducing blood glucose	[65]
Catechin gallate	/	Natural extract	–	Inhibits cardiac hypertrophy	[66]
Cyclic peptide	0.319 µm	Selective inhibitor	Jiajia Liu, School of Pharmacy, Jiangsu University	/	[67]
OSS‐128167	89 µm	Selective inhibitor	Marco Daniele Parenti, University of Bologna	Restricts Hepatitis B Virus Transcription	[68]
SYN	106 µm	Selective inhibitor	Mcule (Budapest, Hungary)	Improved oral glucose tolerance	[69]

## 4 Limitation of the Study

The outcomes of our study present a novel approach for the identification of lysine myristoylated proteins and probe deeply into the crucial role of SIRT6 in CVDs, with particular emphasis on its influence on the myristoylation of ATF2 as well as the resultant effects on endothelial function. Nevertheless, although the developed method for enriching myristoylated peptides exhibits high specificity and efficiency, its applicability across diverse protein types and cellular conditions has not been comprehensively investigated. Further exploration of its performance under varying experimental conditions, cell types, and disease models is essential to guarantee its generalizability. In addition, we may have missed some interesting lysine myristoylation site for its low abundance, and our testing scope does not cover primary endothelial cells, which means that we may omit specific lysine myrsitoylation sites in endothelial cells. Dynamic detection of lysine myristoylation, especially in humans, requires highly specific lysine myrsitoylation antibodies, but the development of antibodies in this field is still in a blank state. Our study's focus on SIRT6's demyristoylation on ATF2 and its implications for endothelial barrier function somewhat restricts its scope. The broader ramifications of lysine myristoylation on other proteins and pathways within CVDs remain largely uncharted territory. Thus, a more exhaustive examination of the functional consequences of myristoylation, encompassing its impacts on protein interaction networks and cellular localization, is imperative. While the study indeed marks a significant progress in comprehending SIRT6's role in endothelial function and introduces a valuable means of identifying myristoylated proteins, additional research is required to tackle the limitations and to fully harness the therapeutic potential of targeting SIRT6 and myristoylation in the context of cardiovascular health.

## 5 Experimental Section

### Cells and Materials

*E.coli* DH5α was bought from Tsingke Biotechnology. *E.coli* ArcticExpress (DE3) was bought from Shanghai Weidi Biotechnology. All peptides were synthesized by Boster Biological Technology. Alkynyl myristic acid (Alk14) was bought from Click Chemistry Tools. The antibody, primers, and peptide sequences were listed below.

**Table:** 

Antibody	Cat	Web
**Beta actin**	ab115777	https://www.abcam.cn/products/primary‐antibodies/beta‐actin‐antibody‐sp124‐cytoskeleton‐marker‐ab115777.html
**SIRT6**	ab191385	https://www.abcam.cn/products/primary‐antibodies/sirt6‐antibody‐epr18463‐ab191385.html
**Biotin**	Invitrogen MA5‐11251	https://www.thermofisher.cn/cn/zh/antibody/product/Biotin-Antibody-Monoclonal/MA5-11251
**ATF2**	**#**35031	https://www.cellsignal.cn/products/primary‐antibodies/atf‐2‐d4l2x‐xp‐rabbit‐mab/35031?site‐search‐type=Products&N=4294956287&Ntt=atf2&fromPage=plp
**Pan‐Myr**	Abbkine ABP57559	https://www.bio‐equip.com/show1equip.asp?equipid=4071544
**Flag**	80010‐1‐RR	DYKDDDDK tag antibody (80010‐1‐RR) | Proteintech | (ptgcn.com)
**VE‐Cadherin**	D87F2	VE‐Cadherin (D87F2) XP® Rabbit mAb | Cell Signaling Technology
**PRKCD**	14188‐1‐AP	PKC Delta antibody (14188‐1‐AP) | Proteintech | (ptgcn.com)
**Primer**	Sequence	
**SIRT6‐F**	GCGCCATGGATCCGGAATTCATGAGCGTTAACTATG	
**SIRT6‐R**	CTAGATTCGAAAGCGGCCGCTTATTTATCATCATCATC	
**pet28aVector‐F**	GCGGCCGCTTTCGAATCTAGAGC	
**pet28aVector‐R**	GAATTCCGGATCCATGGCGCCCTG	
**H133Ymut‐F**	CAGAACTGTATGGTAATATGTTTGTTGAAG	
**H133Ymut‐R**	CATATTACCATACAGTTCTGCCAGTTTATC	
**G60Amut‐F**	CGCAAGCGCTATTCCGGATTTTCGT	
**G60Amut‐R**	CCGGAATAGCGCTTGCGGTGCTAAT	
**S56Ymut‐F**	GGTGCAGGTATTTACACCGCAAGCGGTATTCC	
**S56Ymut‐R**	CGGTGTAAATACCTGCACCGGTATG	
**R65Amut‐F**	CGGATTTTGCTGGTCCGCATGGTGT	
**R65Amut‐R**	GCGGACCAGCAAAATCCGGAATACC	
**Peptides**	**Sequence**	
**B‐H3K9‐5R**	biotin‐KQTARKSTGGKARRRRR	
**B‐H3K9^Ac^‐5R**	biotin‐KQTARK^Ac^STGGKARRRRR	
**B‐H3K9^Myr^‐5R**	biotin‐KQTARK^Myr^STGGKARRRRR	
**B‐H3K9**	biotin‐KQTARKSTGGKA	
**B‐H3K9^Ac^**	biotin‐KQTARK^Ac^STGGKA	
**B‐H3K9^Myr^**	biotin‐KQTARK^Myr^STGGKA	

### Cloning of SIRT6 and Mutant Plasmids

The plasmid pUC57‐SIRT6 contained the full‐length human SIRT6 (1‐355aa) with the flag tag at the C‐terminus. The PCR fragment SIRT6‐Flag from pUC57‐SIRT6 was inserted into pET28a vector to obtain the plasmid pET28a‐SIRT6. Then the pET28a‐SIRT6G60A, pET28a‐SIRT6R65A, pET28a‐SIRT6S56Y, and pET28a‐SIRT6H133Y expression plasmids were obtained through site‐directed mutagenesis by PCR. These SIRT6 expression plasmids were used for SIRT6 expression and purification in *E.coli*.

### Purification of SIRT6 Protein and Mutants

The expression plasmids were transformed in the *E.coli* Arctic Express (DE3) cells, respectively. The *E.coli* cells were cultured at 37 °C in 2X YT culture medium. When OD_600_ reached 0.8, cells were induced by 0.2 mM isopropyl‐1‐thio‐β‐D‐galactopyranoside (IPTG) for 18–20 h at 16 °C. Then, cells were harvested by centrifugation at 6000 rpm for 10min at 4 °C and resuspended in lysis buffer (20 mm Tris‐HCl pH 7.2, 500 mm NaCl, 2% glycerol). Cells were lysed by a high‐pressure homogenizer (Avestin), and centrifuged at 15 000 rpm for room temperature min at 4 °C. The supernatants were incubated with Ni^+^ Sepharose (GE Healthcare) which was pre‐equilibrated in lysis buffer. The resin was washed with 20 column volumes of lysis buffer supplemented with 50 mm imidazole, and then the recombinant proteins were eluted with lysis buffer supplemented with 500 mm imidazole. The eluted fractions were pooled, buffer exchanged (20 mm Tris‐HCl pH 7.2, 250 mm NaCl, 10% glycerol), and concentrated. Finally, the endotoxin was removed by the Pierce High‐Capacity Endotoxin Removal Spin Columns. The purified proteins were stored at −80 °C.

### Determination of the Protein Activities of SIRT6 and Mutants In Vitro

The peptides B‐H3K9‐5R (biotin‐KQTARKSTGGKARRRRR), B‐H3K9^Ac^‐5R (biotin‐KQTARK^Ac^STGGKARRRRR), and B‐H3K9^Myr^‐5R (biotin‐KQTARK^Myr^STGGKARRRRR) were synthesized. Five arginine residues were added to ensure that the peptides were soluble in water. SIRT6 protein SIRT6 and mutants (6 µm) were incubated with the peptides (450 µm) in the reaction system (50 µm Tris‐HCl pH 8.0, 6 mm MgCl_2_, 1 mm NAD^+^) at 30 °C. After 4 h, the reactions were terminated by adding 4% TFA at 37 °C for 10 min. After centrifuging at 12 000rpm for 15 min at room temperature, the supernatants were collected and analyzed via HPLC using an Aeris peptide XB‐C18 column (150 × 4.6 mm, 3.6 µm; Phenomenex, USA). The mobile phase consisted of solvent A (0.1% TFA in HPLC‐grade H2O) and solvent B (0.1% TFA in HPLC‐grade acetonitrile). Peptides were eluted in a linear gradient of 0–90% solvent B at 1 ml min^−1^ over 19 min. The column temperature was set at 25 °C, and the peptides were monitored with UV light (220 nm).

### Biolayer Interferometry Assays

To determinate the affinity of SIRT6 proteins to myristoylated peptides, BLI assays were performed in 96 well plates using an Octet Red96 System. Assays were performed in PBS‐T buffer (1X PBS, 0.05% tween‐20), 120 µM B‐H3K9^Myr^‐5R or B‐H3K9‐5R was attached reversibly to the sensor chip surface. Then, the analytes (SIRT6/SIRT6 mutants/flag peptide, ranged from 0.18 to 5.5 µm) were injected over the sensor surface. We can determine the protein‐peptide affinity by calculating the KD value by the instrument system. Meanwhile, the protein‐peptide affinities were also determined after adding several cofactors (5 mm), such as Mg^2+^, Zn^2+^, ATP, ADP, AMP, cAMP, NAD^+^.

### Co‐Immunoprecipitation Enrichment of B‐H3K9^Myr^‐5R Peptides

SIRT6/mutants (10 µm) were incubated with B‐H3K9^Myr^‐5R or B‐H3K9‐5R (7 µm) in different IP buffers based on buffer (1X PBS, 0.2% tween‐20) at 30 °C for 4h. Then the mixtures were incubated with magnetic flag‐beads at room temperature for 2h. Then, tubes were placed on a magnetic rack for 1 minute, and the supernatants were discarded. The beads were washed three times with an IP buffer and eluted with different elution buffers. The eluants and beads were then analyzed by western blotting with biotin monoclonal antibody or SIRT6 antibody.

### Western Blotting

Protein samples were separated by SDS/PAGE and transferred to the PVDF membrane. The membrane was blocked with 5% BSA in TBST (25 mm Tris, pH 7.4, 150 mm NaCl, 0.1% Tween‐20), incubated with primary antibodies and secondary antibodies in TBST, and then treated with ECL Plus western blotting detection reagents (Bio‐rad). The chemiluminescence signal was analyzed by ChemiDoc imaging system (Bio‐rad).

### RNA Extraction and Quantitative Real‐Time PCR (qPCR)

Total RNA extracted from cells using TriZol Total RNA Isolation Kit (Sangon Biotech, China) was reverse transcribed into cDNA using Prime Script RT Master Mix (Takara Biotechnology, Japan). Then, qPCR was performed on CFX Connected Real‐Time PCR Detection System (Bio‐Rad, USA), using Maxima SYBR Green/Rox qPCR Master Mix (Thermo Fisher Scientific). Fold changes in gene expression in the samples were normalized to β‐actin and calculated by the ΔΔCT method (2−^∆∆Ct^).

### Dual‐Luciferase Reporter Assay

The transcriptional regulation of PRKCD by SIRT6 and ATF2 was assessed using a dual‐luciferase reporter system. 293T cells were seeded in 24‐well plates at 1.5×10^5^cells per well ^−1^ and cultured until 70–80% confluence. Cells were co‐transfected with the following plasmid combinations using Lipofectamine 3000 (Invitrogen): PGL3‐BASIC‐H‐PRKCD promoter construct (firefly luciferase reporter), pRL‐TK control plasmid (Renilla luciferase, Promega) or G60A oe; ATF2 wt oe; ATF2 kr oe. After 24 h, transfected cells were treated with 10 µg mL^−1^ ALK14 or control (0.1% DMSO) for 12 h. Cells were lysed with Passive Lysis Buffer, and firefly/Renilla luciferase activities were quantified using the Dual‐Luciferase Reporter Assay System. Firefly luminescence signals were normalized to Renilla values for each sample.

### Cell Culture

HMEC‐1, obtained from Shanghai Zhong Qiao Xin Zhou Biotechnology, was cultured in MCDB131 medium containing 10% foetal bovine serum (FBS) and 1% antibiotics (penicillin and streptomycin) in 5% CO2 incubator at 37 °C. HUVEC, obtained from Shanghai Zhong Qiao Xin Zhou Biotechnology, were cultured in ECM medium containing 5% FBS, 1% antibiotics (penicillin and streptomycin) and 1% endothelial cell growth supplement (ECGS) in 5% CO2 incubator at 37 °C. HEK 293T were obtained from ATCC, and cultured in Dulbecco's modified Eagle's medium (DM EM) containing 10% FBS and 1% antibiotics (penicillin and streptomycin) in 5% CO2 incubator at 37 °C. The SIRT6 KO 293T cells were constructed by Hanbio Biotechnology, the SIRT6 gRNA was 5′‐GTACGTCCGAGACACAGTCG‐3′. SIRT6 overexpression and knockdown recombinant lentivirus were constructed by Hanbio Biotechnology, the vector pGLV3‐harboring SIRT6 and SIRT6 shRNA (5′‐GCTACGTTGACGAGGTCATGA‐3′) were used for lentivirus packaging. The plasmid ATF2WT, ATF2K296R, GFP‐ATF2WT and GFP‐ATF2K296R were synthesized by Hanbio Biotechnology, and the plasmid pUC57‐SIRT6 and all primers were synthesized by Tsingke Biotechnology.

### Sample Processing

When collecting samples for peptide enrichment, SIRT6 KO 293T cells were cultured to a density of about 80% in high‐glucose DMEM medium and then changed to low‐glucose (1g mL^−1^) DMEM medium culture with 10 µg mL^−1^ Alk14 for 12h. The cells were collected and resuspended in urea lysis buffer (8M urea, 0.1M Tris‐HCl, pH 8.5). Cells were lysed by ultrasonic on ice (100W, 10s working, 10s interval, 10 cycles). The supernatants were taken after centrifugation at 14 000 g for 30 min to obtain whole‐cell protein samples. Samples were quantified by the Bradford method. The SDS‐PAGE electrophoresis and coomassie bright blue staining were used to identify the protein extraction quality. Samples were added with DTT to a final concentration of 10mm, cooled to room temperature for 2h. Then, add IAA to the final concentration of 50mm, and avoid light for 30 min. Added water to dilute the concentration of UA to 1.5m. Samples were digested by trypsin at a 50:1 (protein to trypsin) ratio for about 18h at 37 °C. The peptides were desalted by SPE C18 column (Waters, WAT051910) and lyophilized. The lyophilized peptides were stored at −80 °C.

### Enrichment of Myristoylated Peptides

The lyophilized peptides (1 mg) were redissolved in 400 µL pre‐cooled Myr‐IP buffer (1X PBS, 0.2% tween‐20, 20% acetonitrile) in 1.5mL EP tube, then added 80 µL SIRT6H133Y (1mg  mL^−1^) and 20µL acetonitrile. The mixtures were incubated at 30 °C for 4h. 50 µL anti‐Flag magnetic beads were washed 3 times with Myr‐IP buffer, then discarded the liquid and added into SIRT6H133Y‐peptides mixtures for enrichment reaction. The beads and SIRT6H133Y‐peptides were incubated at room temperature for 2h. Then, beads were precipitated by magnetic rack and discarded the supernatant, 500 µL Myr‐IP buffer was added for washing. After washing three times, remove Myr‐IP buffer completely and add 150 µL of Myr‐elution buffer (0.1 m glycine, 20% acetonitrile, pH 2.2), incubating on ferris wheel at room temperature for 5 min. Repeated the elution 3 times and combined the supernatant from each elution. 4% TFA was added to the elution products and incubated at 37 °C for 10min, after centrifuged at 12 000 rpm for 15min, the supernatants were lyophilized and stored at −80 °C.

### Mass Spectrometric Analysis

The enzymatic hydrolyzed peptides were redissolved in Myr‐MS buffer (0.1% formic acid, 20% acetonitrile) and separated by HPLC using EASY‐nLC 1200 and analyzed with a Q Exactive HF‐X mass spectrometer. The mobile phase A was 0.1% formic acid in water, and mobile phase B was 0.1% formic acid in water (80% acetonitrile). The column was first equilibrated with 100% mobile phase A, and then the peptides were injected by an autosampler to the loading column (2 cm, ID100 µm, 3 µm, C18) and then separated by the analytical column (15 cm, ID150 µm, 1.9 µm, C18) at a flow rate of 600 nL min^−1^. Liquid phase gradient settings were 0 to 5min, 4% to 20% mobile phase B (B); 5 to 110 min, 20% to 45% B; 110 to 112min, 45% to 100% B; and 112 to 120 min, 100% B and the flow rate is 600 nL min^−1^. Peptide samples were separated by analytical columns and analyzed with mass spectrometer. The detection method was positive ion mode, the precursor scanning range was 300–1400 m/z, the resolution of MS1 was 120 000 at 200 m/z, the AGC (Automatic gain control) target was 3e6, the Maximum IT was 30ms, and the Dynamic exclusion time was 12.0 s. The m/z of peptides and peptide fragments were acquired as follows: 60 fragment profiles (MS2 scans) were acquired after each full scan, using HCD fragmentation mode, Normalized Collision Energy was 27%, Isolation window was 1.6 m/z, and MS2 resolution was 7500 at 200 m/z.Raw data (RAW files) collected by MS analysis were retrieved through the database using Proteome Discoverer (version 2.3.0.523) with a Mascot (version 2.3.01) to obtain protein information for samples. The search parameters were set as follows: enzyme was Trypsin; database was Human_refseq (27414 proteins, version 04/07/2013); missed cleavage sites set to 2; dynamically modifications were N‐terminal acetylation, Oxidation (M), and ALK14 labeling (K) (m/z 234.377, C16H26O); Peptide Mass Tolerance, 20ppm; Fragment Mass Tolerance, 50mmu. The minimum peptide length was set at 6. The false discovery rate (FDR) of peptides and proteins was set at 1%.

### 4D‐Label‐Free Proteomic Analyses

ATF2 overexpression (WT), ATF2 K296R overexpression (KR), and empty control (Ctrl) plasmids were transfected to HMEC‐1 lines. After treatment of ALK14 (10 µg mL^−1^) for 12 h. Cells were lysed by trypsin and centrifuged at 12 000 rpm for 10 min. Next the supernatant was discarded, and the Sediment was stored at −80 °C. Further proteomic analysis was conducted by Shanghai iProteome Biotechnology Co, Ltd. HPLC was performed by system EASY‐nLC 1200 with a nanoliter flow rate was used to separate samples, with the mobile phase A was 0.1% formic acid aqueous solution, mobile phase B is 0.1% formic acid acetonitrile aqueous solution (acetonitrile is 80%). After chromatographic column separation, peptides were analyzed using Q Exact HF‐X mass spectrometer. The detection method is positive ion mode, with the ion scanning range from 300 to 1400m/z. The threshold for differential proteins was (|log2 (Fold change) |≥0.2, and *p* <0.05).

### Bioinformatic Analysis

The GO enrichment analysis was performed on Metascape (https://metascape.org/gp/index.html#/main/step1). Subcellular localization of myristoy‐modified protein was annotated on Uniprot (https://www.uniprot.org/uploadlists/) using pie chart. Protein function predictions on five different PPI datasets (STRING, BioGrid, OmniPath, InWeb, IM) were performed by metascape (https://metascape.org/gp/index.html#/main/step1). Motif analysis for lysine myristoylation substrates‐Programs Motif‐X (MoMo – Submission form meme‐suite.org) were used to analyze the model of identified myristoylation sequences of myristoyl‐21‐mers (10 amino acids upstream and downstream of the myristoylation sites). The expression of PRKCD was analyzed in different tissues of the cardiovascular system using the Genotype‐Tissue Expression (http://gtexportal.org/) database. To predict the 3D protein structure, Protein Data Bank (PDB, http://www.rcsb.org/), AlphaFold Protein Structure Database (https://alphafold.com/) were used, and the results were compared. Pymol (https://pymol.org/2/) was used to display images.

### Click IT

Click chemistry was used for the detection of K296 myristoylation of exogenous ATF2. The click chemistry‐based labeling and detection technology was conducted according to the manufacturer's instructions (Click‐iT Protein Reaction Buffer Kit Invitrogen C10276).

### Chromatin Immunoprecipitation Quantitative PCR (ChIP‐qPCR)

293T and HUVEC cells were cultured to 80% confluence in complete medium. Cells were transfected with ATF2 wt oe plasmid or empty vector control using Lipofectamine 3000 (Invitrogen) according to manufacturer's protocol. After 48‐hour incubation, parallel samples were fixed with formaldehyde to stabilize the interactions between chromatin proteins and DNA. Then we conducted chromatin immunoprecipitation to enrich DNA fragments with chromatin proteins. Following reverse Cross‐linking and DNA purification, the qPCR was used to measure the enriched DNA. We utilized the reported PRKCD promoter sequence as reported.^[^ ^55^ ^]^

### Transendothelial Permeability Assays

HMEC‐1 transfected with relative plasmids were pretreated with ALK14(10 µg mL^−1^) for 12 h and were grown on 0.4 µm‐pore transwell filters (Millipore, Bedford, MA) until confluent. Fluorescein isothiocyanate (FITC)‐dextran (#FD2000S, #FD70S, Sigma‐Aldrich, St. Louis, MO) solution at 6µg mL^−1^ was then added into the upper chamber and allowed for incubation for 2 h or 6h. Fluorescence of media in the lower chamber was detected at 485/530 nm by afluorescence microplate reader (FL × 800, BIO‐TEK Instruments).

### Generation of Endothelial‐Specific *Sirt6*‐Knockout Mice

Endothelial specific Sirt6 KO (ec*Sirt6^−/−^*) mice (on a C57/Bl6J background) were developed by Cyagen Biosciences lnc. and litter of wild‐type (WT) mice served as control. Genotyping by tail preparation and PCR were performed at 2 weeks of age. All animal procedures were conducted under animal welfare guidelines and were approved by the Animal Ethics Committee of Zhongshan Hospital, Fudan University (EARIA‐389).

### Caudal Vein Injection with Adeno‐Associated Virus

One hundred microliters of AAV‐Tie‐ATF2‐ZsGreen, AAV‐Tie‐ATF2K298R‐EGFP viruses, bought from Hanbio Biotechnology (Shanghai, China), at a density of 5 × 10^12^ v.g. mL^−1^ were injected into 4‐week‐old ec*Sirt6* ^−/−^ mice via the caudal vein.

### Blood Lipid Measurement

The serum lipid profiling protocol was adapted from established methods described in ref. [56]

### In Vivo Vascular Permeability Assay

The relative AAVS were injected into 8 weeks male endothelial specific SIRT6^−/–^ mice 4 weeks before collecting ascending and descending aortas. A high‐fat diet was conducted 2 weeks after injection in high fat groups. The permeability of mouse aortas was assessed in vivo by using Evans blue, the classic tracers that transport across the epithelial/endothelial monolayer through the paracellular pathway to indicate the barrier function. The Evans blue dye (3% solution in 1×PBS, 50 µL per mouse) was intravenously injected through angular vein. After 5 min, mice were perfused with 4% paraformaldehyde from the heart, and then aortas were quickly harvested. Images of the whole aortas were captured, and the distribution of Evans blue was further determinedby ImageJ software. Evans blue dye was eluted from the aortas by incubation with formamide at 56 °C for 2 days. The amount of dye was quantified by spectrophotometry at 610 nm.

### Mouse Aortic Endothelial Cells (MAECs) Isolation

Mouse aortic endothelial cells (MAECs) were isolated from mouse aorta according to a previously described method and cultured in Endothelial Cell Medium.^[^ ^30^ ^]^

### Histological Immunofluorescence Staining

Following saline perfusion, aortas underwent fixation in 4% paraformaldehyde for 24 h prior to paraffin embedding. Samples were sectioned at 5 µm thickness using a microtome. For immunofluorescence, deparaffinized and rehydrated slides underwent antigen retrieval through 30‐minute incubation in 98 °C retrieval buffer. After cooling and washing, sections received overnight incubation at 4 °C with primary antibodies diluted in blocking buffer (TBS containing 5% horse serum and 1% BSA). Following primary antibody application, sections were washed and treated with secondary antibodies for 60 min at room temperature. Slides were coverslipped using DAPI‐containing ProLong Gold mounting medium. Imaging utilized Leica confocal microscopy systems.

### En Face Immunofluorescence (IF) Staining

The cultivation and collection of mice were described above. Remove the outer membrane and longitudinally cut the thoracic aorta of mice for surface staining. After being fixed in 4% paraformaldehyde for 20 min, 0.5% Triton X‐100 was infiltrated into PBS for 30 min, and 10% normal goat serum was blocked in PBS for 1 h. Then, the corresponding primary antibody was incubated overnight at 4 °C. Then, wash HUVECs or tissues three times with PBS, coupled with the corresponding Alexa Fluor 488‐conjugated goat anti‐rabbit IgG (Thermo, cat#A‐11008) or Alexa Fluor 594 ‐conjugated goat anti‐mouse IgG (Thermo, cat#A‐11 005) (1:500; at room temperature, place in a closed solution for 1 h). Cover the slide with DAPI containing fluorine shielding adhesive medium. Randomly select representative images from each group. Fluorescence signal detection was performed using Leica confocal laser scanning microscope (Leica SP8).

### Statistical Analysis

Non‐normal data were log‐transformed prior to analysis. Data from this study are presented as the mean ± S.E.M of at least three replicates. Experimental ‘n = ’ denotes biological replicates in figure legends. The statistical significance of the difference between different groups was determined by two‐tailed unpaired Student's *t*‐test or one‐way ANOVA, as applicable. *p* < 0.05 was considered statistically significant (*), while *p* > 0.05 was considered not significant (ns). All analyses performed in GraphPad Prism.

## Conflict of Interest

The authors declare no conflict of interest.

## Author Contributions

R.F., Z.F., S.Z., Y.Z., N.W., Z.G., X.Z., B. J., and H.X. contributed equally to this work. H.L., L.H., Y.Z., P.L., and J.G. proposed the conception and study design and had the final approval of the manuscript submitted; G.Z., W.Z., H.L., Y.H., S.Q., H.L., X.W., and J.Z. gave good suggestions for improving the manuscript; R.F., Z.F., S.Z., Y.Z., N.W., Z.G., X.Z., B.J., and H.X. participated in the data collection and analysis, the drafting of the manuscript and the submission; H.H., Z.Z., M.A., X.Y., J.G., X.N., S.A., D.H., H.L., L.S., X.Z., Z.C., J.G., H.Z., Y.H., S.W., J.P., J.S., and X.W. participated in the data collections and a few additional works.

## Supporting information



Supporting Information

Supporting Information

## Data Availability

The data that support the findings of this study are available from the corresponding author upon reasonable request.
