# Supplementary material for: SIRT6 Lysine‐Demyristoylates ATF2 to Ameliorate Vascular Injury via PRKCD/VE‐Cadherin Pathway Regulating Vascular Endothelial Barrier
Source: Adv Sci (Weinh). 2025 Aug 14;12(41):e04948. doi: 10.1002/advs.202504948 (PMC12591191; doi:10.1002/advs.202504948)
Supplement: Supplementary file 1 — Supporting Information [file ADVS-12-e04948-s001.pdf]

Supplementary Figures and Legends

Figure S1. Binding Affinity of SIRT6 Mutant to H3K9^{Myr} in the Presence of Mg^{2+}.

(**A**) Biolayer Interferometry (BLI) assay showing the binding affinity of H133Y mutant to B-H3K9^{Myr}-5R with Mg2+ supplementation. Optical thickness, measured in nanometers (nm), was recorded on the SA biosensor layer. The equilibrium binding signal (Req) was identified by a plateau in the response curve. (**B**) BLI assay for S56Y mutant's binding to B-H3K9^{Myr}-5R, performed under standard conditions and with Mg^{2+}. The optical thickness (nm) on the SA biosensor layer indicates response levels. A plateau in the curve denotes the achievement of Req. (**C**) BLI assay evaluating the binding affinity of R65A mutant to B-H3K9^{Myr}-5R, with assays conducted under standard conditions and with Mg^{2+}. The assay's response, measured as optical thickness (nm) on the SA biosensor, leads to the determination of Req through a plateau in the response curve. (**D**) BLI assay to assess the binding affinity of G60A mutant to B-H3K9^{Myr}-5R under standard conditions. The optical thickness (nm) on the SA biosensor layer reflects response levels, with Req identified by a leveling off in the curve. (**E**) A statistical table summarizing the binding affinity of various SIRT6 mutants and peptide segments under different experimental conditions. "nt" signifies no detection.

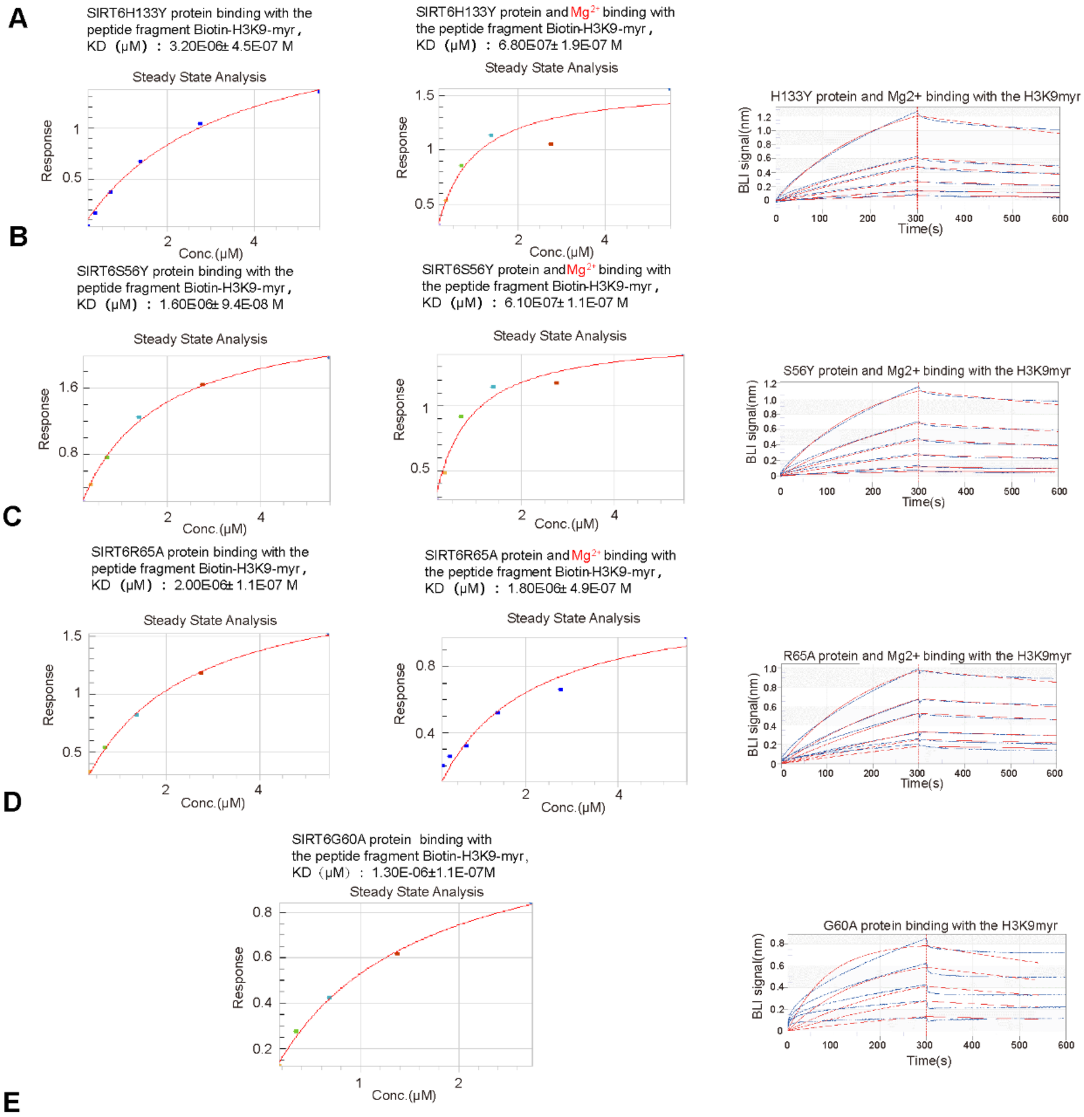

E

Peptide	Protein	K_D(μM)	Peptide	Protein	K_D(μM)
	Flag	nt		SIRT6	nt
	SIRT6	8.10±0.96			
	H133Y	3.20±0.45		H133Y	nt
	H133Y+Mg^{2+}	0.68±0.19			
B-H3K9^{Myr}-5R	S56Y	1.60±0.094	B-H3K9-5R	S56Y	nt
	S56Y+Mg^{2+}	0.61±0.11			
	R65A	2.0±0.11		R65A	nt
	R65A+Mg^{2+}	1.80±0.49			
	G60A	1.30±0.11		G60A	nt

Figure S2. Binding Affinity of SIRT6 Mutant and FLAG to H3K9 and H3K9^{Myr}.

(**A**) BLI assay measuring the binding affinity of SIRT6 to B-H3K9-5R under standard conditions, with optical thickness (nm) on the SA biosensor layer indicating response levels. (**B-F**) BLI assays evaluating the binding affinity of S56Y, H133Y, R65A, G60A mutants, and FLAG to their respective targets under standard conditions. For each, the optical thickness (nm) on the SA biosensor layer provides the response measure.

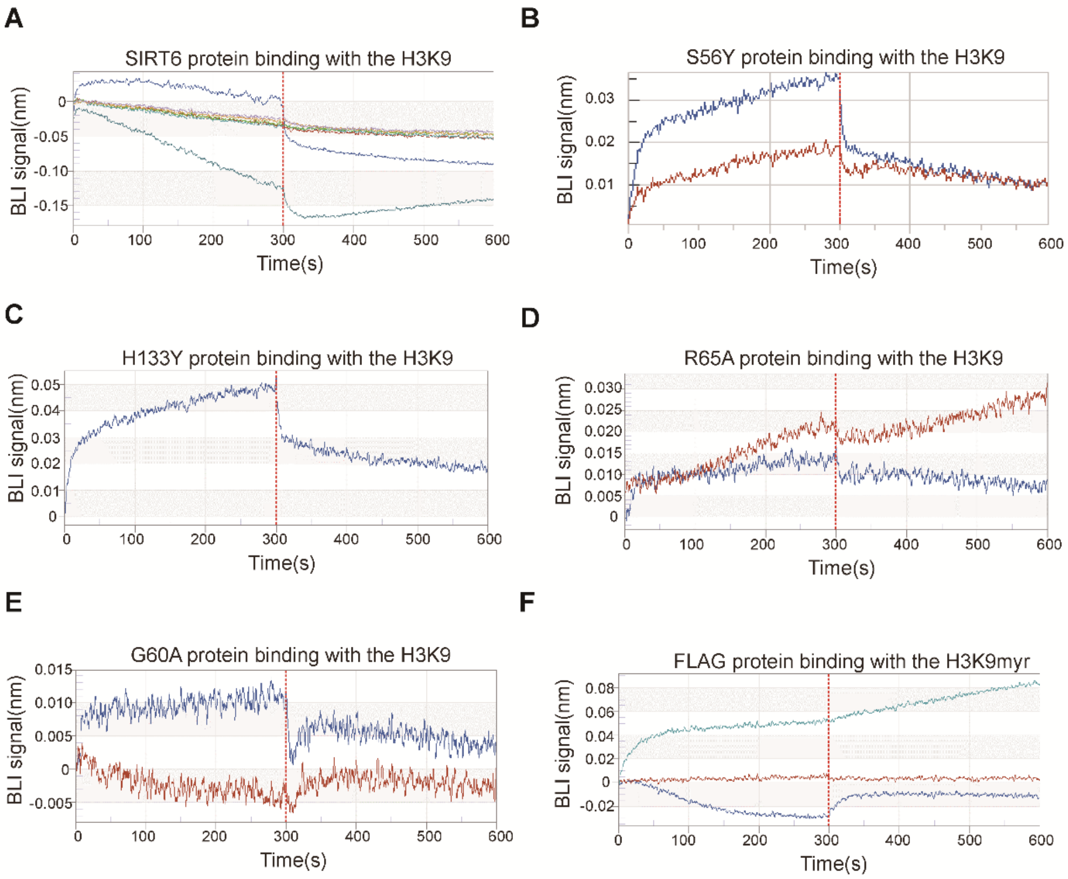

A
SIRT6 protein binding with the H3K9
BLI signal(nm)
Time(s)
B
S56Y protein binding with the H3K9
BLI signal(nm)
Time(s)
C
H133Y protein binding with the H3K9
BLI signal(nm)
Time(s)
D
R65A protein binding with the H3K9
BLI signal(nm)
Time(s)
E
G60A protein binding with the H3K9
BLI signal(nm)
Time(s)
F
FLAG protein binding with the H3K9myr
BLI signal(nm)
Time(s)

Figure S3. Binding Affinity of H133Y to H3K9^{Myr} with Single or Multiple Cofactors.

(**A**) BLI assay of H133Y's binding to B-H3K9^{Myr}-5R in the presence of a single cofactor, with response indicated by optical thickness (nm) on the SA biosensor layer. (**B**) BLI assay for H133Y's affinity to B-H3K9^{Myr}-5R amidst multiple cofactors, measured similarly. (**C**) Confirming the specificity of H133Y by using Co-Immunoprecipitation (Co-IP) for the enrichment of negative control and myristoylated peptides to H133Y, including system selection and reaction conditions. The histogram represented the relative Biotin level (n=3). Data were presented as mean ± S.E.M and analyzed using a two-tailed t-test.

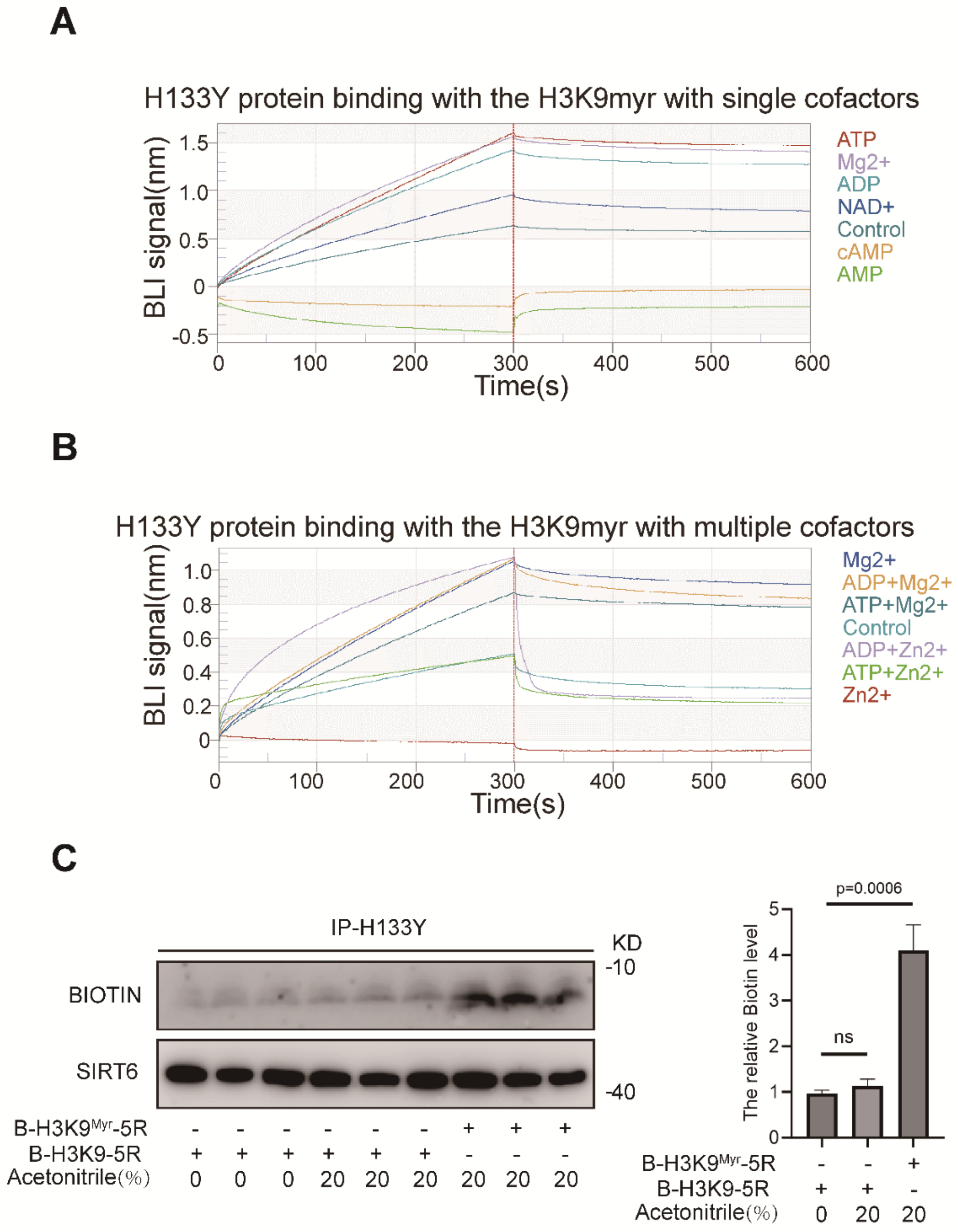
A
H133Y protein binding with the H3K9myr with single cofactors
BLI signal(nm)
Time(s)
ATP
Mg2+
ADP
NAD+
Control
cAMP
AMP
B
H133Y protein binding with the H3K9myr with multiple cofactors
BLI signal(nm)
Time(s)
Mg2+
ADP+Mg2+
ATP+Mg2+
Control
ADP+Zn2+
ATP+Zn2+
Zn2+
C
IP-H133Y
KD
BIOTIN
SIRT6
-10
-40
B-H3K9Myr-5R
B-H3K9-5R
Acetonitrile(%)
The relative Biotin level
p=0.0006
ns

Figure S4. Verification of ALK14-Mimic Myristoylation.

(**A**) Western blot analysis showing global cell myristoylation levels in SIRT6 KO 293T cells treated with a gradient of ALK14 (0-10 μg/ml) for 12 hours. (**B**) Western blot analysis of pan myristoylation levels in KO or WT groups treated with or without ALK14 (10 μg/ml). (**C**) Immunoprecipitation (IP) assays were performed in the lysates of SIRT6 KO 293T cells to detect the acetylation level of ATF2. Before lysis, cells were transfected with SIRT6 mutants and treated with ALK14 (10ug/ml) for 12 hours. The histogram represented the relative acetylation level of ATF2 (n=3). (**D**) Western blot analysis of NMT1 level in HUVECs transfected with SIRT6 and ctrl plasmids. The histograms show the relative protein level (%) (n=3). Data were presented as mean ± S.E.M and analyzed using a two-tailed t-test.

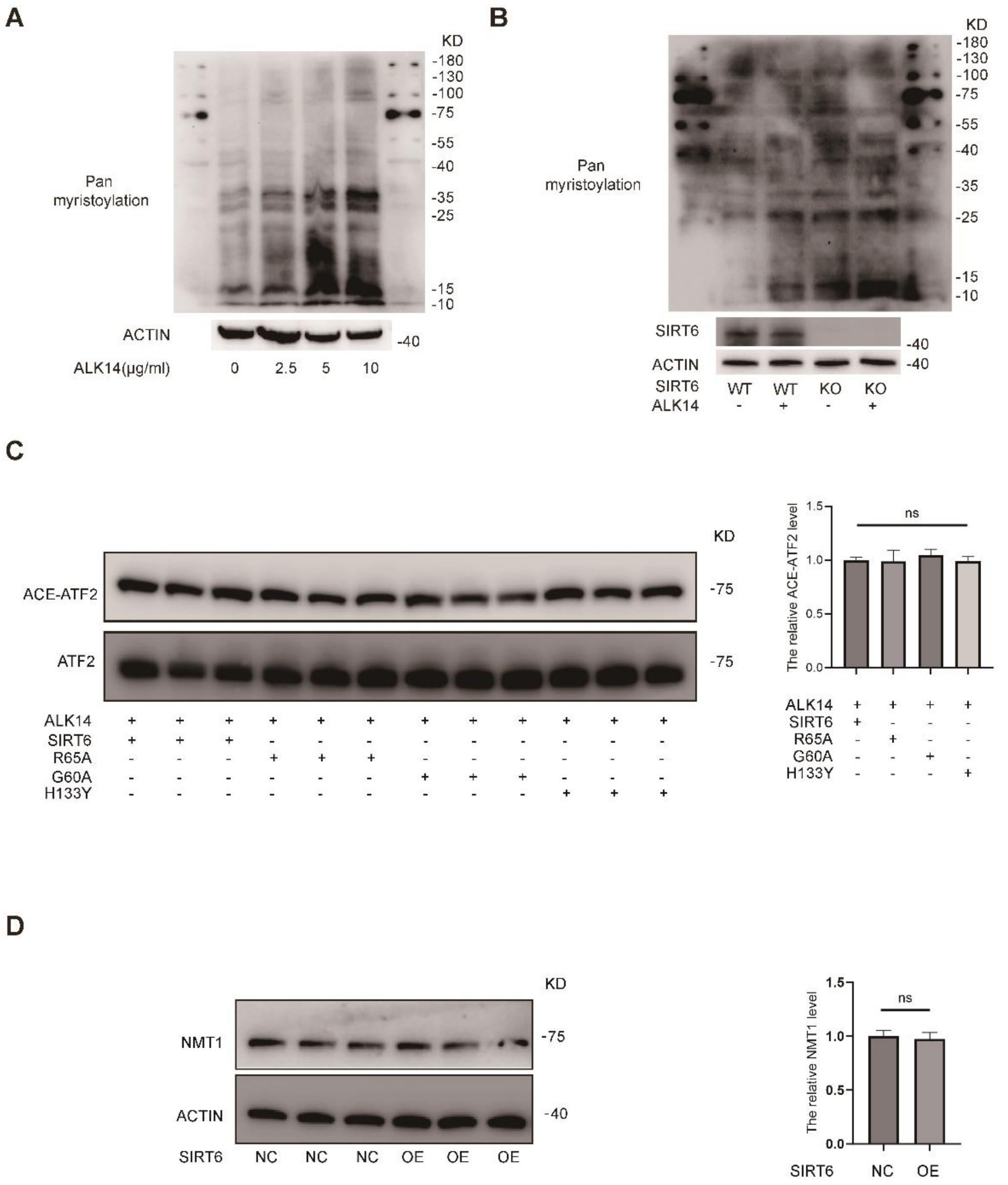
A
KD
-180
-130
-100
-75
-55
-40
-35
-25
-15
-10
Pan myristoylation
ACTIN
-40
ALK14(μg/ml)
0
2.5
5
10
B
KD
-180
-130
-100
-75
-55
-40
-35
-25
-15
-10
Pan myristoylation
SIRT6
-40
ACTIN
-40
SIRT6 WT WT KO KO
ALK14 - + - +
C
KD
ACE-ATF2
-75
ATF2
-75
ALK14 + + + + + + + + + + + +
SIRT6 + + + - - - - - - - - -
R65A - - - + + + - - - - - -
G60A - - - - - - + + + - - -
H133Y - - - - - - - - - + + +
The relative ACE-ATF2 level
1.5
1.0
0.5
0.0
ns
ALK14 + + + +
SIRT6 + - - -
R65A - + - -
G60A - - + -
H133Y - - - +
D
KD
NMT1
-75
ACTIN
-40
SIRT6 NC NC NC OE OE OE
The relative NMT1 level
1.5
1.0
0.5
0.0
ns
SIRT6 NC OE

Figure S5. Wild type ATF2 and K296R mutants plasmids transformation and IP related evidence.

(**A**) Representative fluorescence plots displaying the efficiency of ATF2WT or ATF2 K296R plasmid transfection into 293T SIRT6KO or WT cells treated with or without ALK14 (10 µg/ml) for 12 hours (n=3). (**B**) Immunoprecipitation enrichment of exogenous ATF2 and its mutants in the absence of ALK14 treatment (n=3). (**C, D**) Immunoprecipitation performed after ALK14 treatment (10 µg/ml for 12 hours) in 293T SIRT6KO or WT cells to enrich exogenous ATF2 and its mutants. (**E**) Schematic illustration of constructing pcDNA3.1-CMV-ATF2-3xflag-EF1-ZsGreen-T2A-Puro and pcDNA3.1-CMV-ATF2K296R-3xflag-EF1-ZsGreen-T2A-Puro plasmids. (**F**) Diagram showing the construction of pHBAAV-TIE-Atf2K298R-3xflag-T2A and pHBAAV-TIE-Atf2-3xflag-T2A adenoviruses. (**G**) Immunoprecipitation (IP) assays were performed in the lysates of SIRT6 KO 293T to detect the acetylation level of ATF2. Before lysis, cells were transfected with ATF2-FLAG (WT) and ATF2 K296R-FLAG (KR) plasmids. Histogram showing the relative acetylation level of ATF2 (n=3). Date is represented as means ± S.E.M. P value by two-tailed t-test (**B, C, D, G**).

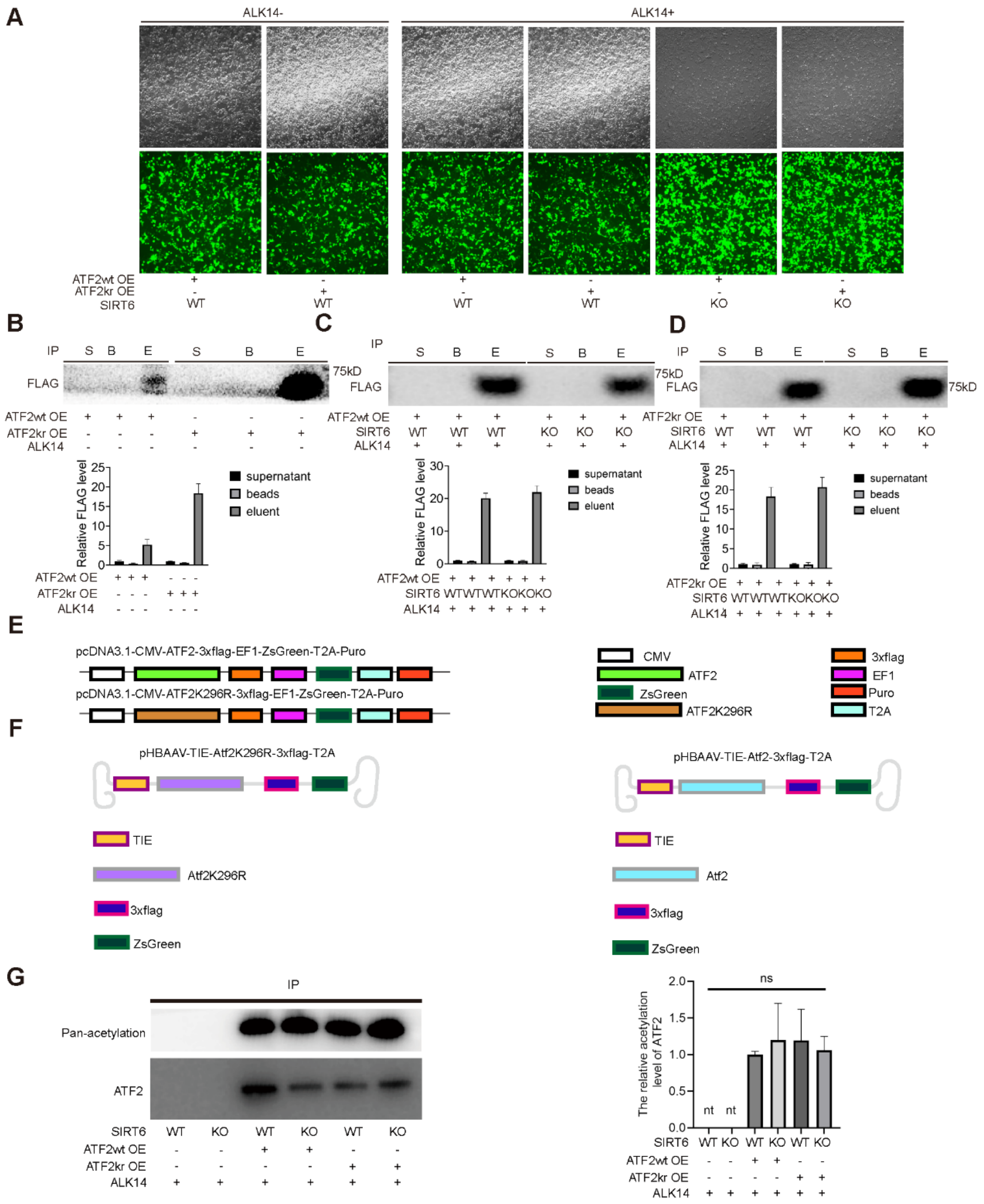
A
ALK14-
ALK14+
ATF2wt OE
ATF2kr OE
SIRT6
WT
KO
B
IP
S B E
FLAG
75kD
supernatant
beads
eluent
Relative FLAG level
ATF2wt OE
ATF2kr OE
ALK14
C
D
E
pcDNA3.1-CMV-ATF2-3xflag-EF1-ZsGreen-T2A-Puro
pcDNA3.1-CMV-ATF2K296R-3xflag-EF1-ZsGreen-T2A-Puro
CMV
ATF2
ZsGreen
ATF2K296R
3xflag
EF1
Puro
T2A
F
pHBAAV-TIE-Atf2K296R-3xflag-T2A
pHBAAV-TIE-Atf2-3xflag-T2A
TIE
Atf2K296R
Atf2
3xflag
ZsGreen
G
Pan-acetylation
ATF2
ns
nt
The relative acetylation level of ATF2

Figure S6. ATF2 nuclear cytoplasmic distribution.

(**A**) Nucleoplasmic separation and western blot assays were performed in SIRT6 KO cells to detect the level of ATF2 in cytoplasm and nucleus. Before lysis, cells were transfected with WT and KR plasmids under ALK14 (10μg/ml) treatment for 12 hours or not. The histograms show the relative ATF2 level (%) in nuclear (n=3). (**B**) Immunofluorescence staining of ATF2 (green) in each group under ALK14 treatment or not. DAPI was used for counterstaining cellular nuclei (blue) (n =10, scale bars =20 μm). (**C**) Nucleoplasmic separation and western blot assays were performed in SIRT6 KO and WT cells to detect the level of ATF2 in cytoplasm and nucleus. Before lysis, cells were transfected with WT and KR plasmids under ALK14 (10μg/ml) treatment for 12 hours. The histograms show the relative ATF2 level (%) in nuclear (n=3). (**D**) Immunofluorescence staining of ATF2 (green) in each group under ALK14 treatment. DAPI was used for counterstaining cellular nuclei (blue) (n =10, scale bars =20 μm). (**E**) Nucleoplasmic separation and western blot assays were performed in primary HUVEC cells to detect the level of ATF2 in cytoplasm and nucleus. Before lysis, cells were transfected with SIRT6 overexpression and control adenovirus under ALK14 (10μg/ml) treatment for 12 hours or not. The histograms show the relative ATF2 level (%) in nuclear (n=3). The data are presented as the means ± s.e.m. P values were calculated by t-test (**A, C, E**).

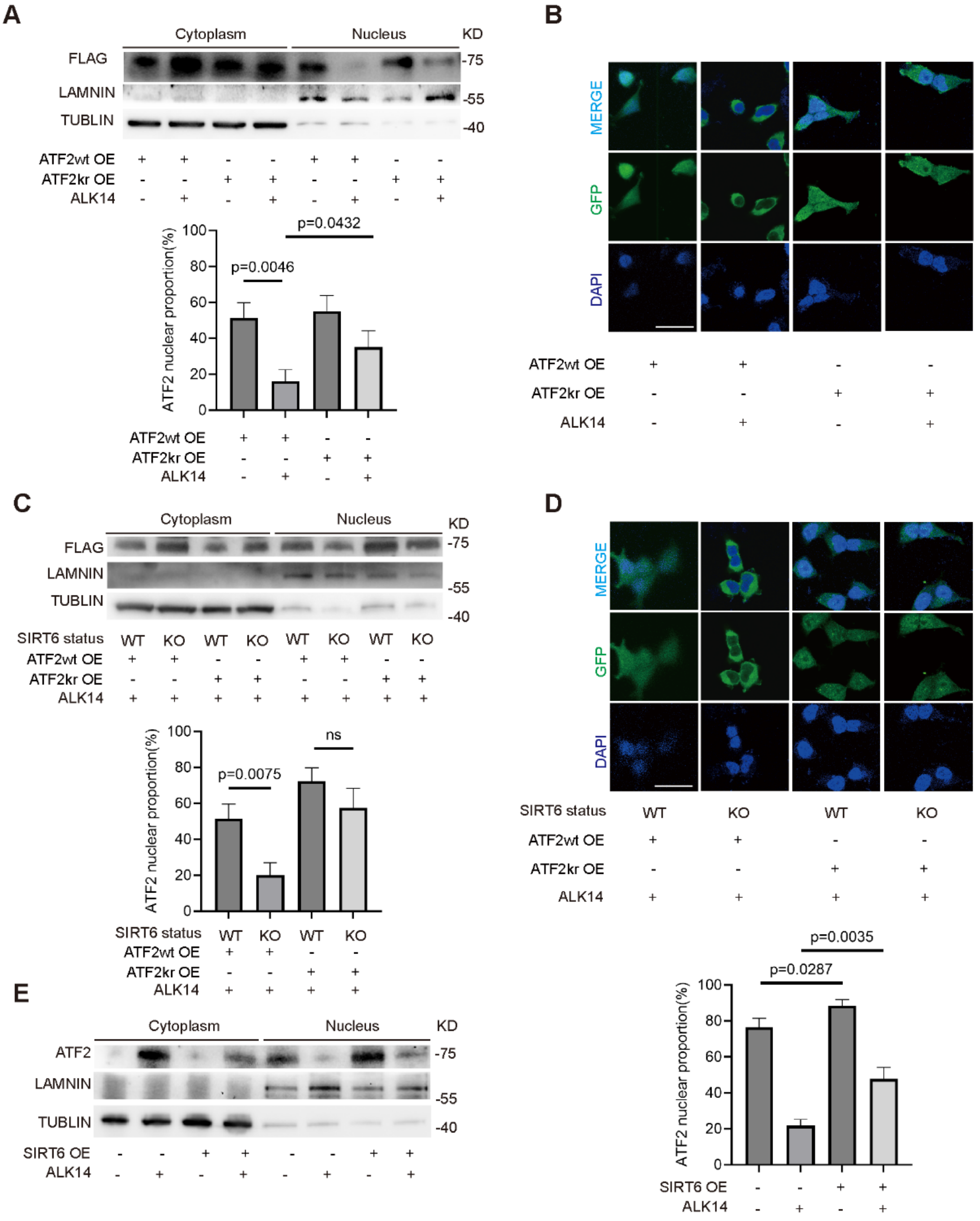
A
Cytoplasm
Nucleus
KD
FLAG
LAMNIN
TUBLIN
-75
-55
-40
ATF2wt OE
ATF2kr OE
ALK14
ATF2 nuclear proportion(%)
p=0.0046
p=0.0432
B
MERGE
GFP
DAPI
ATF2wt OE
ATF2kr OE
ALK14
C
Cytoplasm
Nucleus
KD
FLAG
LAMNIN
TUBLIN
SIRT6 status
WT KO WT KO WT KO WT KO
ATF2wt OE
ATF2kr OE
ALK14
ATF2 nuclear proportion(%)
p=0.0075
ns
D
MERGE
GFP
DAPI
SIRT6 status
WT KO WT KO
ATF2wt OE
ATF2kr OE
ALK14
p=0.0035
p=0.0287
ATF2 nuclear proportion(%)
SIRT6 OE
ALK14
E
Cytoplasm
Nucleus
KD
ATF2
LAMNIN
TUBLIN
SIRT6 OE
ALK14

Figure S7. Repeat images of Fig.3 D-F

(**A**) Nucleoplasmic separation and western blot assays were performed in SIRT6 KD HMEC cells to detect the level of ATF2 in cytoplasm and nucleus. Before lysis, cells were transfected with ATF2 WT and KR plasmids under ALK14 (10μg/ml) treatment for 12 hours or not. (**B**) Nucleoplasmic separation and western blot assays were performed in SIRT6 KD HMEC cells to detect the level of ATF2 in cytoplasm and nucleus. Before lysis, cells were transfected with G60A, ATF2 WT and KR plasmids under ALK14 (10μg/ml) treatment for 12 hours. (**C**) Nucleoplasmic separation and western blot assays were performed in SIRT6 KD HMEC cells to detect the level of ATF2 in cytoplasm and nucleus. Before lysis, cells were transfected with G60A and ATF2 WT plasmids under ALK14 (10μg/ml) treatment for 12 hours or not.

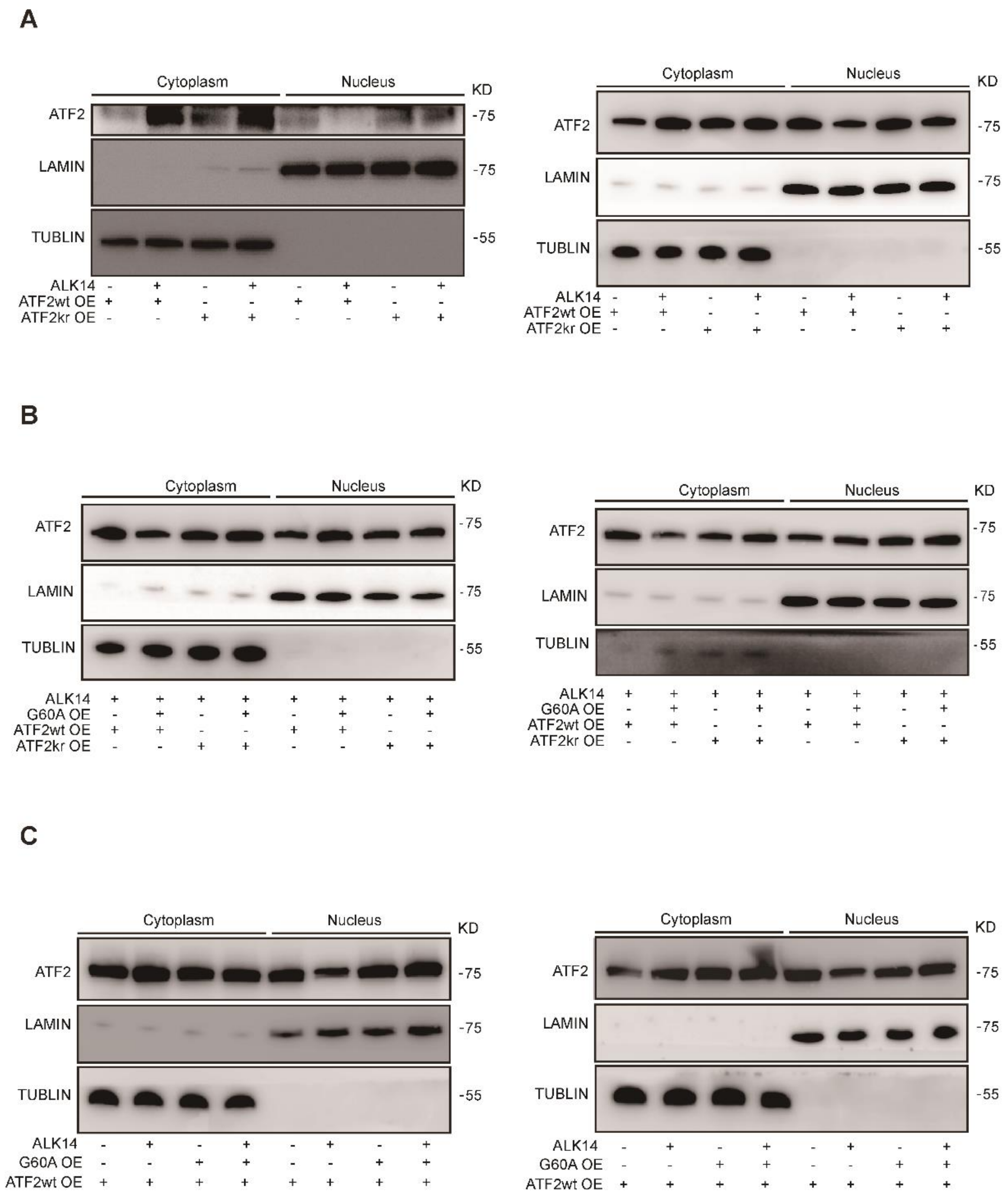
A
B
C
Cytoplasm
Nucleus
KD
ATF2
LAMIN
TUBLIN
-75
-55
ALK14
ATF2wt OE
ATF2kr OE
G60A OE

Figure S8. Phenotypes related to endothelial barrier function.

(**A**) Western blot analysis of VE-Cadherin, FLAG, PRKCD level in HMEC-1 transfected with ATF2-FLAG overexpression (WT) and ATF2 K296R-FLAG overexpression (KR) plasmids treated with or without ALK14 (10 ug/ml) for 12h (n=3). (**B**) Western blot analysis of VE-Cadherin, PRKCD, SIRT6 level in HMEC-1 transfected with SIRT6 overexpression (OE) and Ctrl (OENC) Lentivirus with or without ALK14 treatment (n=3). (**C**) Western blot analysis of VE-Cadherin, PRKCD, SIRT6 level in SIRT6 overexpression HMEC-1 and its control transfected with siPRKCD with ALK14 treatment (n=3). (**D**) The histograms represented the quantitative assays of endothelial permeability in WT and KR HMEC-1 with or without ALK14 treatment (n=3). (**E**) The histograms represented the quantitative assay of endothelial permeability in SIRT6 overexpression HMEC-1 and its control with or without ALK14 treatment (n=3). (**F**) The histogram represented the quantitative assay of endothelial permeability of SIRT6 overexpression HMEC-1 and its control transfected with siPRKCD with ALK14 treatment (n=3). (**G**) Western blot analysis of CD31 and αSMA level in HMEC-1 transfected with ATF2-FLAG overexpression (WT) and ATF2 K296R-FLAG overexpression (KR) plasmids treated with or without ALK14 (10 ug/ml) for 12h (n=3). The date is represented as means ± S.E.M. P value by two-tailed t-test (**A, B, C, D, E, F, G**).

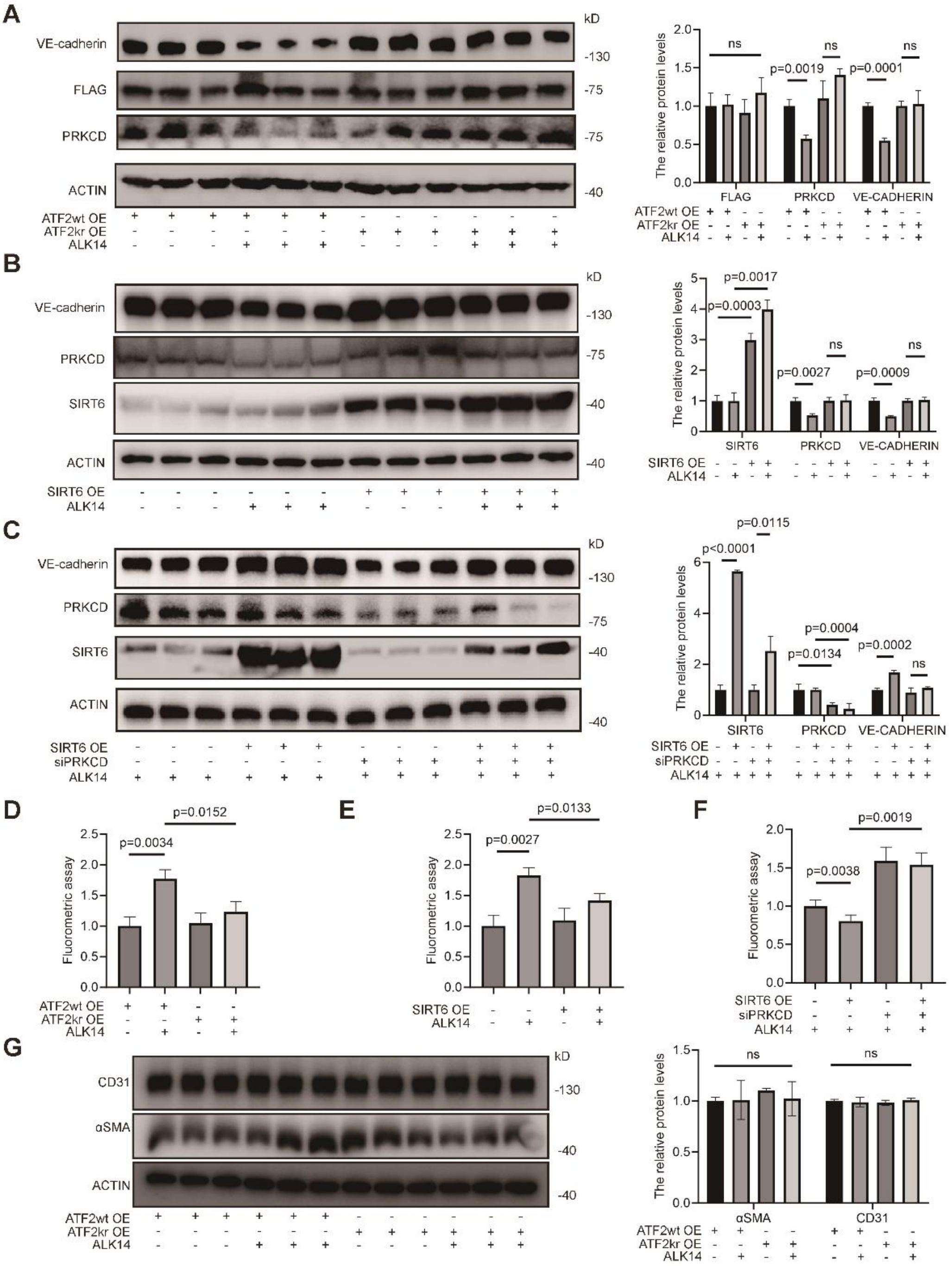
A
VE-cadherin
FLAG
PRKCD
ACTIN
kD
-130
-75
-75
-40
ATF2wt OE
ATF2kr OE
ALK14
The relative protein levels
ns
p=0.0019
ns
p=0.0001
ns
FLAG
PRKCD
VE-CADHERIN
B
VE-cadherin
PRKCD
SIRT6
ACTIN
kD
-130
-75
-40
-40
SIRT6 OE
ALK14
The relative protein levels
p=0.0017
p=0.0003
ns
p=0.0027
p=0.0009
ns
SIRT6
PRKCD
VE-CADHERIN
C
VE-cadherin
PRKCD
SIRT6
ACTIN
kD
-130
-75
-40
-40
SIRT6 OE
siPRKCD
ALK14
The relative protein levels
p=0.0115
p<0.0001
p=0.0004
p=0.0134
p=0.0002
ns
SIRT6
PRKCD
VE-CADHERIN
D
Fluorometric assay
p=0.0152
p=0.0034
ATF2wt OE
ATF2kr OE
ALK14
E
Fluorometric assay
p=0.0133
p=0.0027
SIRT6 OE
ALK14
F
Fluorometric assay
p=0.0019
p=0.0038
SIRT6 OE
siPRKCD
ALK14
G
CD31
αSMA
ACTIN
kD
-130
-40
-40
ATF2wt OE
ATF2kr OE
ALK14
The relative protein levels
ns
ns
αSMA
CD31

Figure S9. Immunofluorescence display of endothelial barrier.

(**A**) Immunofluorescence staining analyses of VE-Cadherin (green) in HMEC-1 transfected with ATF2 WT overexpression (WT), ATF2 296R overexpression (KR) plasmids with or without ALK14 (10 μg/ml) treatment for 12 hours. Nuclei were counterstained with DAPI (blue) (n =8-10, scale bars =50 μm). The histograms show the distribution of the intercellular 5um or 10um gaps of each group (n=8-10). (**B**) Immunofluorescence staining analyses of VE-Cadherin (green) in SIRT6 overexpression and its control HMEC-1 (constructed by lentivirus) transfected with WT and KR plasmids with ALK14 treatment (n=8-10, scale bars=50 μm). The histograms show the distribution of intercellular 5um or 10um gaps of each group (n=8-10). (**C**) Immunofluorescence staining analyses of VE-Cadherin (green) in SIRT6 overexpression and its control HMEC-1 transfected with siPRKCD with ALK14 treatment (n =8-10, scale bars =50 μm). The histograms show the distribution of the intercellular 5um or 10um gaps of each group (n=8-10). Data were presented as mean ± S.E.M and analyzed using a two-tailed t-test (**A-C**). ns indicates no significant difference.

A

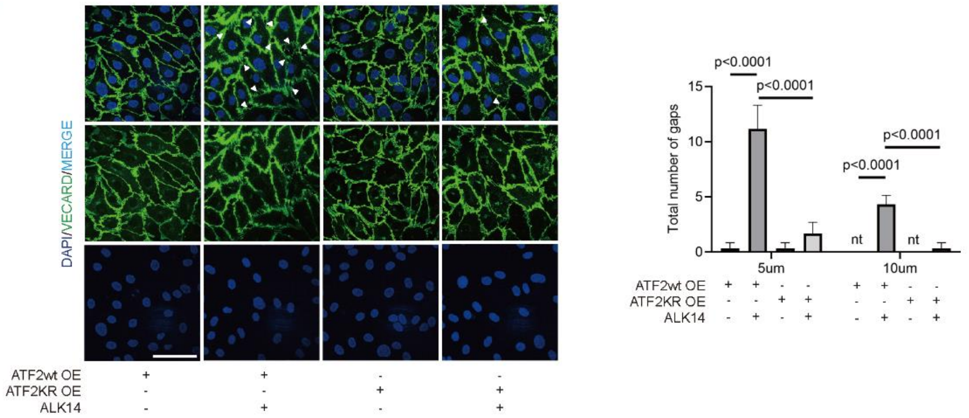
DAPI/VECARD/MERGE
ATF2wt OE + + - -
ATF2KR OE - - + +
ALK14 - + - +
Total number of gaps
p<0.0001
p<0.0001
p<0.0001
p<0.0001
nt
nt
5um
10um
ATF2wt OE
ATF2KR OE
ALK14

B

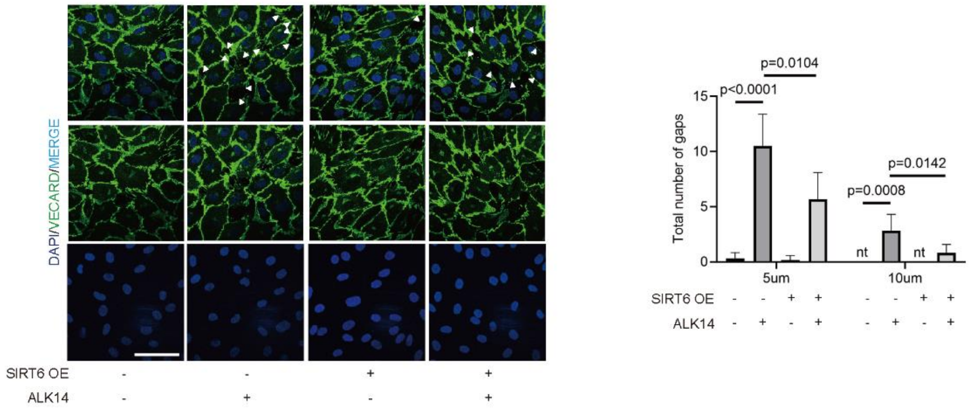
DAPI/VECARD/MERGE
SIRT6 OE - - + +
ALK14 - + - +
Total number of gaps
p<0.0001
p=0.0104
p=0.0008
p=0.0142
nt
nt
5um
10um
SIRT6 OE
ALK14

C

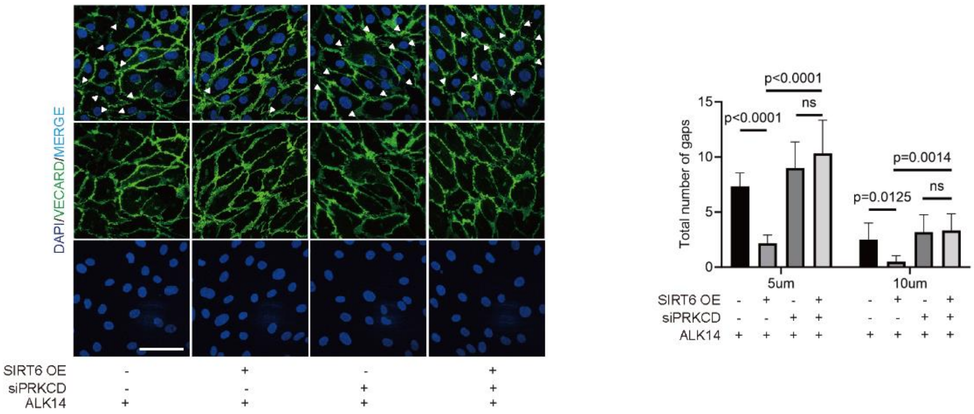
DAPI/VECARD/MERGE
SIRT6 OE - + - +
siPRKCD - - + +
ALK14 + + + +
Total number of gaps
p<0.0001
p<0.0001
ns
p=0.0125
p=0.0014
ns
5um
10um
SIRT6 OE
siPRKCD
ALK14

Figure S10 ALK14 and natural myristic acid comparation

(**A**) Nucleoplasmic separation and western blot assays were performed in HUVEC cells to detect the level of ATF2 in cytoplasm and nucleus. Before lysis, cells were treated with ALK14 (10μg/ml) or Myristic acid (10μg/ml) for 12 hours or not. (**B**) The histograms show the relative ATF2 level (%) in nuclear. (**C**) Immunofluorescence staining analyses of VE-Cadherin (red) and ATF2(green) in HUVECs treated with ALK14 (10μg/ml) or Myristic acid (10μg/ml) for 12 hours or not. (**D**) The histograms (up) show the ATF2 nuclear proportion. The histograms show the distribution of the intercellular 5um or 10um gaps of each group (down). scar bar= 100μm (n=6-8) (**E**) Western blot analysis of VE-Cadherin, PRKCD level in HUVECs treated with ALK14 (10 ug/ml) or MA (10 ug/ml) for 12h or not. (**F**)The histograms show the relative protein level (%) (n=3). Data were presented as mean ± S.E.M and analyzed using a two-tailed t-test. ns indicates no significant difference.

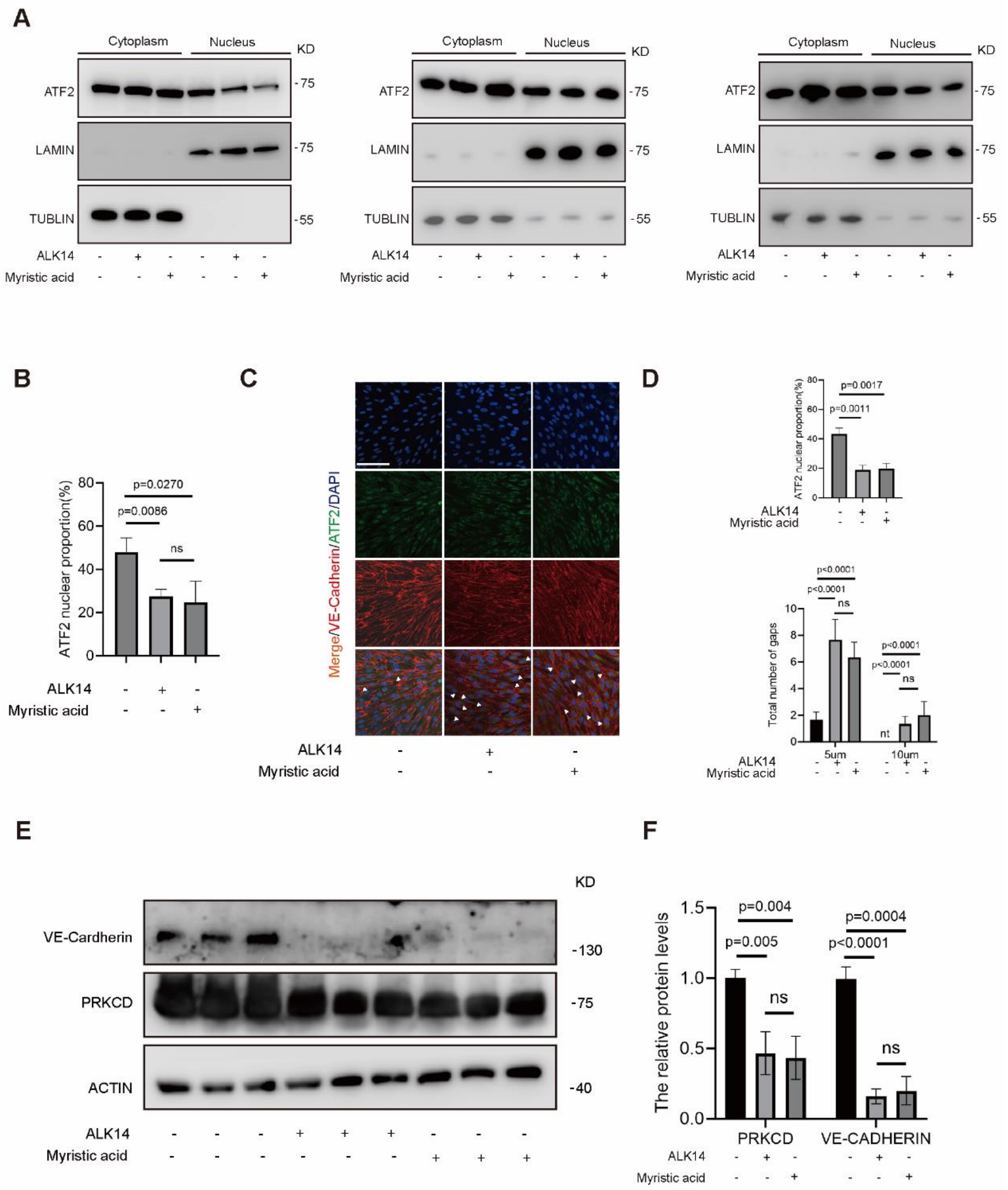

A
Cytoplasm
Nucleus
KD
ATF2
LAMIN
TUBLIN
-75
-55
ALK14
Myristic acid
B
ATF2 nuclear proportion(%)
p=0.0270
p=0.0086
ns
C
Merge/VE-Cadherin/ATF2/DAPI
D
p=0.0017
p=0.0011
Total number of gaps
p<0.0001
ns
nt
5um
10um
E
VE-Cardherin
PRKCD
ACTIN
-130
-40
F
The relative protein levels
p=0.004
p=0.005
p=0.0004
p<0.0001
PRKCD
VE-CADHERIN

Figure S11. Regulation of ATF2 transcription by PRKCD and Generation of ecSIRT6-/- Mice.

(**A**) The dual luciferase assay was used to 293T cells transfected with G60A and ATF2 plasmids without ALK14 treatment. The histogram represented the PRKCD-luc activity (Firefly/Renilla ratio) (n=6). (**B**) The dual luciferase assay was used to 293T cells transfected with ATF2 WT and KR plasmids with or without ALK14 treatment. The histogram represented the PRKCD-luc activity (Firefly/Renilla ratio) (n=6). (**C**) The dual luciferase assay was used to 293T cells transfected with G60A plasmids with or without ALK14 treatment. The histogram represented the PRKCD-luc activity (Firefly/Renilla ratio) (n=6). (**D**) Immunoprecipitation (IP) assays were performed in the lysates of SIRT6 KO and WT 293T to detect the acetylation level and myristoylation level of PRKCD. Before lysis, cells were treated with ALK14 (10ug/ml) for 12 hours. The histogram represented the relative myristoylation or acetylation level of PRKCD (n=3). (**E**) A ChIP-qPCR experiments confirmed ATF2 as an upstream transcription factor regulating the PRKCD gene expression (n=3). (**F**) Schematic representation of conditional knockout mice generation with Sirt6 ablation in endothelial cells using the Cre-LoxP recombination system. Deletions were made in exons 3, 4, 5, and 6 via Cdh5-Cre-mediated recombination. **(G)** Tables (up) and histogram (down) represented the blood cholesterol (TC), triglyceride (TG), low-density lipoprotein (LDL-C), and high-density lipoprotein (HDL-C) levels in mice fed normally or HFD for 2 weeks (n=6). (**H**) The RT-qPCR analyses of ATF2 mRNA level in each group after collection (n=6). (**I**) Western blot analyses of ATF2, SIRT6 expression in each group (n=6). The histogram showed the relative ATF2, SIRT6 expression (n=6). Data were presented as mean ± S.E.M and analyzed using two-tailed t-test (**A-I**).

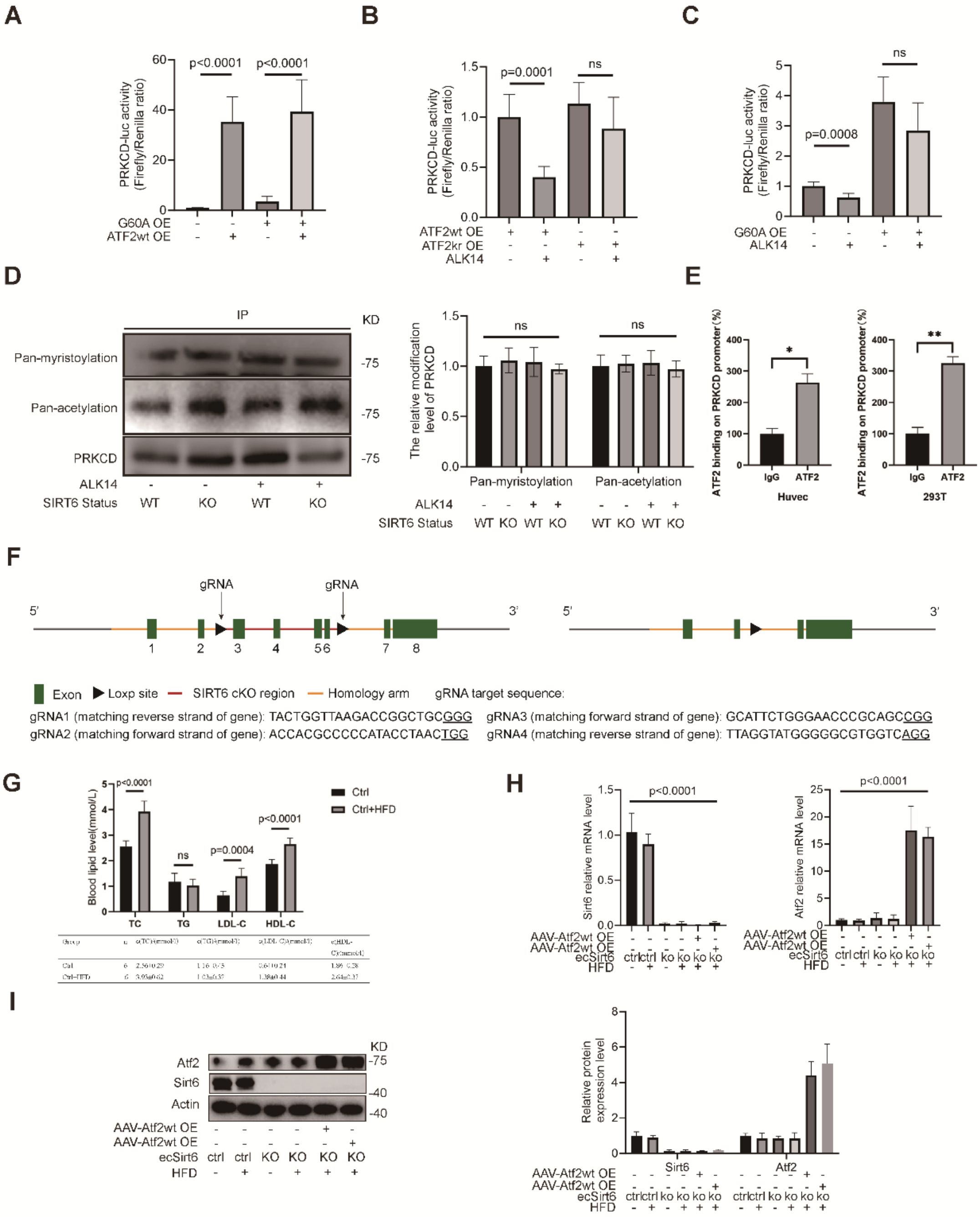
A
PRKCD-luc activity (Firefly/Renilla ratio)
p<0.0001
G60A OE
ATF2wt OE
B
p=0.0001
ns
ATF2wt OE
ATF2kr OE
ALK14
C
p=0.0008
D
IP
KD
Pan-myristoylation
Pan-acetylation
PRKCD
SIRT6 Status
The relative modification level of PRKCD
E
ATF2 binding on PRKCD promoter (%)
IgG
ATF2
Huvec
293T
F
gRNA
5'
3'
Exon
Loxp site
SIRT6 cKO region
Homology arm
gRNA target sequence:
gRNA1 (matching reverse strand of gene): TACTGGTTAAGACCGGCTGCGGG
gRNA2 (matching forward strand of gene): ACCACGCCCCCATACCTAACTGG
gRNA3 (matching forward strand of gene): GCATTCTGGGAACCCGCAGCCGG
gRNA4 (matching reverse strand of gene): TTAGGTATGGGGGCGTGGTCAGG
G
Blood lipid level(mmol/L)
Ctrl
Ctrl+HFD
p=0.0004
TC
TG
LDL-C
HDL-C
H
Sirt6 relative mRNA level
Atf2 relative mRNA level
AAV-Atf2wt OE
ecSirt6
HFD
I
Atf2
Sirt6
Actin
Relative protein expression level

Figure S12. The WB repeat of Fig 8K

(**A-B**) Western blot analyses of VE-Cadherin, Prkcd expression in each group (n=6).

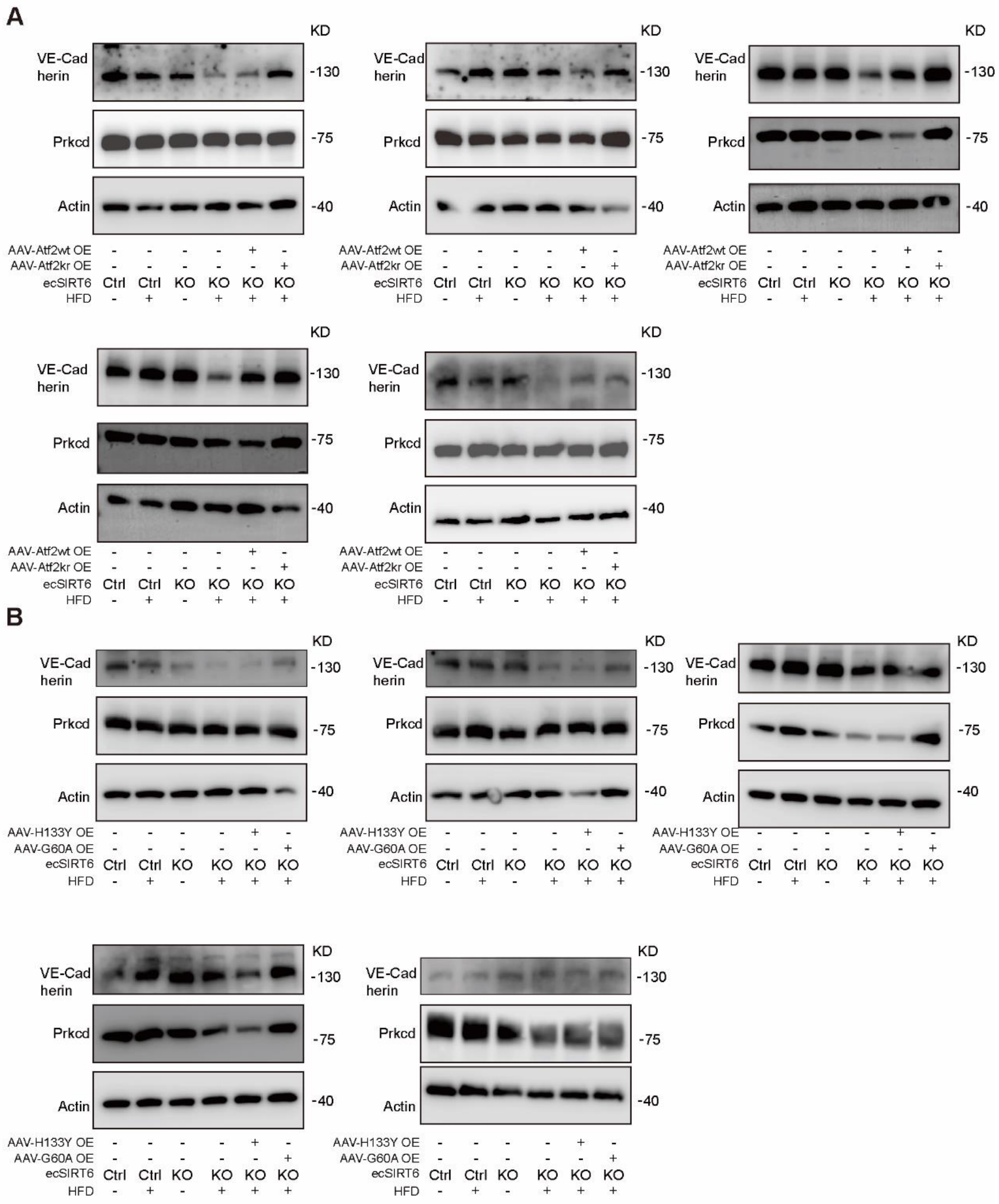
A
KD
VE-Cad herin
-130
Prkcd
-75
Actin
-40
AAV-Atf2wt OE - - - - + -
AAV-Atf2kr OE - - - - - +
ecSIRT6 Ctrl Ctrl KO KO KO KO
HFD - + - + + +
B
KD
VE-Cad herin
-130
Prkcd
-75
Actin
-40
AAV-H133Y OE - - - - + -
AAV-G60A OE - - - - - +
ecSIRT6 Ctrl Ctrl KO KO KO KO
HFD - + - + + +

Figure S13. SIRT6 regulates endothelial barrier function *in vivo*.

(**A**) The schematic illustration showing the *in vivo* tail vein injection of ATF2-overexpression AAV (ATF2WT), ATF2K298R-overexpression AAV (ATF2KR) into 8 weeks male endothelial specific SIRT6 knockout (ecSIRT6$^{-/-}$) mice 4 weeks before collecting ascending and descending aortas. Each group received a specific diet before injection. (**B**) Immunofluorescence staining analyses of VE-Cadherin (red) in the aortic section from different groups. Nuclei were counterstained with DAPI (blue) (n =6-8, scale bars =200 μm). The histograms (down) shows the the relative VE-Cadherin fluorescent intensity level (fold change%). scar bar= 100μm (n=6-8). (**C**) En face Immunofluorescence staining analyses of VE-Cadherin (red) and VCAM-1(green) in the descending aortas of each group. The histograms (up) shows the relative mean junction continuity (fold change%). The histograms (down) shows the the relative VCAM-A fluorescent intensity level (fold change%). scar bar= 50μm (n=6-8). (**D**)The transcription of SIRT6 in human peripheral samples from patients atherosclerotic lesions or control arteries without atherosclerotic lesions (from deceased organ donors) was evaluated using the published GSE 100927 dataset. The y axis shows 104 artery samples divided into the ttherosclerotic and cotrol carotid, femoral and infra-popliteal artery groups. The x axis shows the normalized (and averaged) signal intensity (In) of SIRT6. Data were presented as mean ± S.E.M and analyzed using two-tailed t-test (**B-C**).

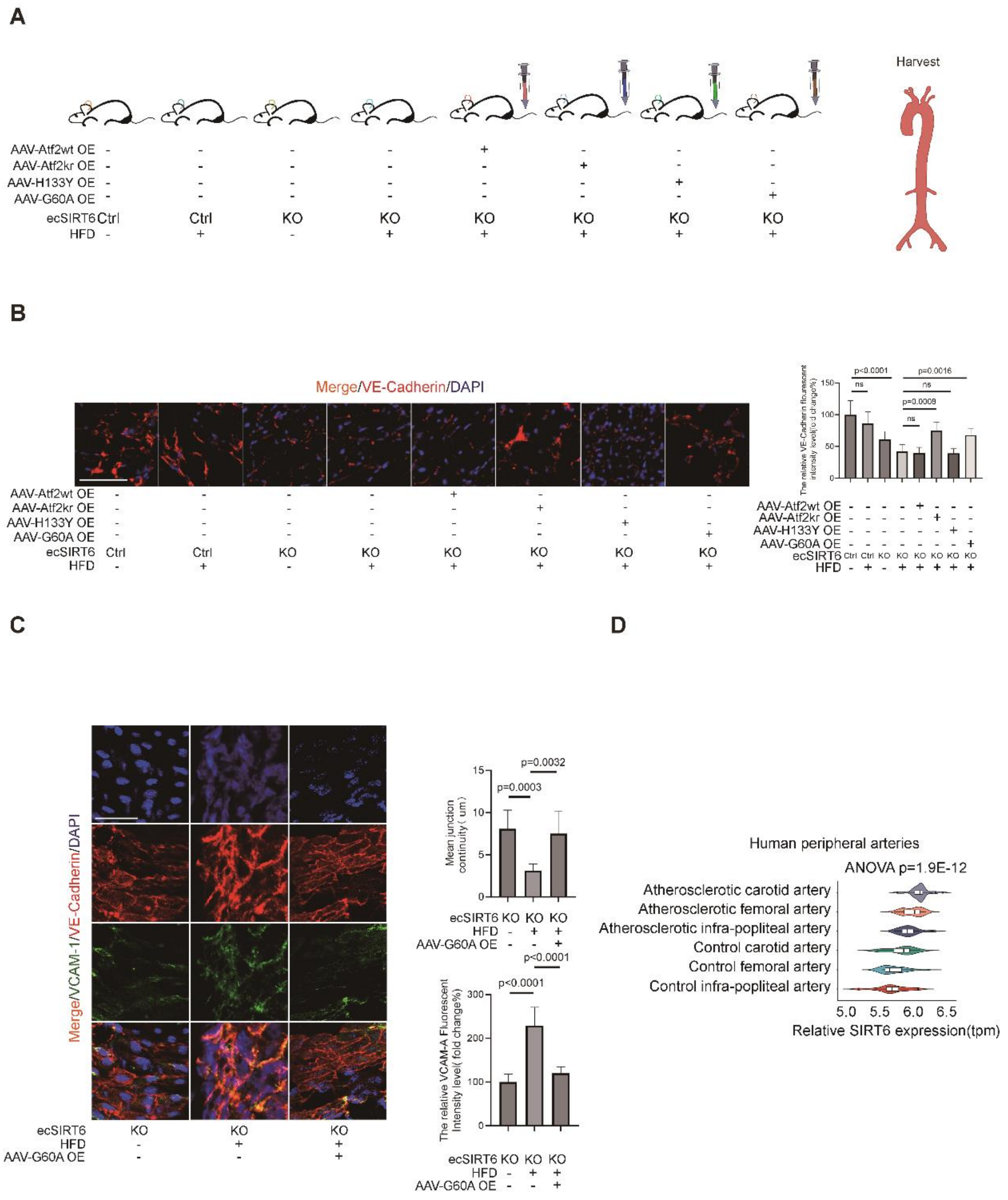
A
Harvest
AAV-Atf2wt OE
AAV-Atf2kr OE
AAV-H133Y OE
AAV-G60A OE
ecSIRT6 Ctrl Ctrl KO KO KO KO KO KO
HFD
B
Merge/VE-Cadherin/DAPI
AAV-Atf2wt OE
AAV-Atf2kr OE
AAV-H133Y OE
AAV-G60A OE
ecSIRT6 Ctrl Ctrl KO KO KO KO KO KO
HFD
p<0.0001
p=0.0016
p=0.0009
ns
C
Merge/VCAM-1/VE-Cadherin/DAPI
ecSIRT6 KO KO KO
HFD
AAV-G60A OE
Mean junction continuity(um)
p=0.0003
p=0.0032
p<0.0001
p<0.0001
The relative VCAM-A Fluorescent Intensity level(fold change%)
D
Human peripheral arteries
ANOVA p=1.9E-12
Atherosclerotic carotid artery
Atherosclerotic femoral artery
Atherosclerotic infra-popliteal artery
Control carotid artery
Control femoral artery
Control infra-popliteal artery
5.0 5.5 6.0 6.5
Relative SIRT6 expression(tpm)
