# Supplementary material for: SIRT6 Lysine‐Demyristoylates ATF2 to Ameliorate Vascular Injury via PRKCD/VE‐Cadherin Pathway Regulating Vascular Endothelial Barrier
Source: Adv Sci (Weinh). 2025 Aug 14;12(41):e04948. doi: 10.1002/advs.202504948 (PMC12591191; doi:10.1002/advs.202504948)
Supplement: Supplementary file 2 — Supporting Information [file ADVS-12-e04948-s002.doc]

**Supplementary Tables**

**Table S1. Representation of the identified lysine myristoylated proteins.**

| **Protein accession** | **Position** | **Amino acid** | **Protein names** | **Sequence** | **Confidence** | **Quality PEP** | **Quality qValue** | **Modifications** | **Gene_symbol** |
| --- | --- | --- | --- | --- | --- | --- | --- | --- | --- |
| P06744 | 130 | K | Glucose-6-phosphate isomerase | VLDKMKSFCQGPLMVTEALKPYSSGGPR | High | 0.00674136 | 0.000181 | 1XALK14 [K6] | GPI |
| P10909 | 57 | K | Clusterin | EIQNAVNGVKQIKTLIEK | High | 0.0482686 | 0.001099 | 1xALK14 [K13] | CLU |
| Q04656 | 1094 | K | Copper-transporting ATPase 1 | ILAIVGTAESNSEHPLGTAITK | High | 0.0834033 | 0.002812 | 1xALK14 [K22] | ATP7A |
| Q9NRL2 | 113 | K | Bromodomain adjacent to zinc finger domain protein 1A | LHEICDDIFAYVK | High | 0.096337 | 0.003588 | 1xALK14 [K13] | BAZ1A |
| Q96HW7 | 140 | K | Integrator complex subunit 4 | SHQVLAQLLDTLLAIGTK | High | 0.150934 | 0.007411 | 1xALK14 [K18] | INTS4 |
| Q5VVQ6 | 214 | K | Ubiquitin thioesterase OTU1 | TNQEYCDWIK | High | 0.170072 | 0.009106 | 1xALK14 [K10] | YOD1 |
| Q12906 | 348 | K | Interleukin enhancer-binding factor 3 | VLGMDPLPSKMPKKPK | High | 0.241479 | 0.006036 | 1xALK14 [K16] | ILF3 |
| Q7Z759 | 173/174 | K | T-complex protein 1 subunit theta | ILGSGISSSSVLHGMVFKK | High | 0.238273 | 0.006036 | 2xALK14 [K18, K19] | CCT8 |
| A0A8J8YUZ5 | 616 | K | Liprin-alpha-4 | MIQEEKESTELR | High | 0.306442 | 0.006909 | 1xALK14 [K6] | PPFIA4 |
| P09619 | 13 | K | Platelet-derived growth factor receptor beta | MRLPGAMPALALK | High | 0.322885 | 0.007864 | 1xALK14 [K13] | PDGFRB |
| P15336 | 296 | K | Cyclic AMP-dependent transcription factor ATF-2 | MRLKAALTQQHPPVTNGDTVK | High | 0.32854 | 0.008695 | 1xALK14 [K21] | ATF2 |
| Q9BZE0 | 315 | K | Zinc finger protein GLIS2 | YTDPSSLRKHIK | High | 0.341588 | 0.008695 | 1xALK14 [K12] | GLIS2 |
| P62328 | 4 | K | Thymosin beta-4 | MSDKPDMAEIEKFDK | High | 0.0725645 | 0.002071 | 1xALK14 [K4] | TMSB4X |
| Q9NP97 | 15 | K | Dynein light chain roadblock-type 1 | LQSQKGVQGIIVVNTEGIPIK | High | 0.10415 | 0.003904 | 1xALK14 [K5] | DYNLRB1 |
| A1X283 | 567 | K | SH3 and PX domain-containing protein 2B | GPTPKPPGVILPMMPAK | High | 0.119459 | 0.004612 | 1xALK14 [k17] | SH3PXD2B |
